# Small-molecule inhibitor of HlyU attenuates virulence of *Vibrio* species

**DOI:** 10.1038/s41598-019-39554-y

**Published:** 2019-03-13

**Authors:** Zee-Won Lee, Byoung Sik Kim, Kyung Ku Jang, Ye-Ji Bang, Suhyeon Kim, Nam-Chul Ha, Young Hyun Jung, Hyun Jik Lee, Ho Jae Han, Jong-Seo Kim, Jeesoo Kim, Pramod K. Sahu, Lak Shin Jeong, Myung Hee Kim, Sang Ho Choi

**Affiliations:** 1 National Research Laboratory of Molecular Microbiology and Toxicology, Department of Agricultural Biotechnology and Center for Food Safety and Toxicology, Seoul National University, Seoul, 08826 South Korea; 2 Department of Food Science and Engineering, Ewha Womans University, Seoul, 03760 South Korea; 3 Infection and Immunity Research Laboratory, Metabolic Regulation Research Center, Korea Research Institute of Bioscience and Biotechnology, Daejeon, 34141 South Korea; 4 Department of Agricultural Biotechnology and Center for Food Safety and Toxicology, Seoul National University, Seoul, 08826 South Korea; 5 Department of Veterinary Physiology, College of Veterinary Medicine, Research Institute for Veterinary Science, and BK21 PLUS Program for Creative Veterinary Science Research, Seoul National University, Seoul, 08826 South Korea; 6 Center for RNA Research, Institute for Basic Science, Seoul National University, Seoul, 08826 South Korea; 7 School of Biological Sciences, Seoul National University, Seoul, 08826 South Korea; 8 Department of Pharmacy, Seoul National University, Seoul, 08826 South Korea; 9 Future Medicine Co., Ltd, Seoul, 06665 South Korea; 10 Present Address: Department of Microbiology, New York University School of Medicine, New York, NY 10016 USA; 11 Present Address: Department of Immunology, The University of Texas Southwestern Medical Center, 6000 Harry Hines Blvd., Dallas, TX 75390 USA

## Abstract

Increasing antibiotic resistance has led to the development of new strategies to combat bacterial infection. Anti-virulence strategies that impair virulence of bacterial pathogens are one of the novel approaches with less selective pressure for developing resistance than traditional strategies that impede viability. In this study, a small molecule CM14 [*N*-(4-oxo-4H-thieno[3,4-c]chromen-3-yl)-3-phenylprop-2-ynamide] that inhibits the activity of HlyU, a transcriptional regulator essential for the virulence of the fulminating human pathogen *Vibrio vulnificus*, has been identified. Without affecting bacterial growth or triggering the host cell death, CM14 reduces HlyU-dependent expression of virulence genes in *V. vulnificus*. In addition to the decreased hemolysis of human erythrocytes, CM14 impedes host cell rounding and lysis caused by *V. vulnificus*. Notably, CM14 significantly enhances survival of mice infected with *V. vulnificus* by alleviating hepatic and renal dysfunction and systemic inflammation. Biochemical, mass spectrometric, and mutational analyses revealed that CM14 inhibits HlyU from binding to target DNA by covalently modifying Cys30. Remarkably, CM14 decreases the expression of various virulence genes of other *Vibrio* species and thus attenuates their virulence phenotypes. Together, this molecule could be an anti-virulence agent against HlyU-harboring *Vibrio* species with a low selective pressure for the emergence of resistance.

## Introduction

Traditional strategies to combat bacterial infection are mostly dependent on the use of antibiotics that inhibit bacterial viability. However, inhibition of viability leads to the inevitable emergence of strains resistant to antibiotics. The emergence and spread of antibiotic-resistant bacteria have become a threat to public health by reducing the effectiveness of present antibiotics, and thus these are a major cause for the rising healthcare costs^1–3^. This situation leads to an imminent need for the development of new strategies to impede the virulence, rather than viability, of bacterial pathogens^4,5^. Anti-virulence strategies disarm the pathogens, thereby rendering them harmless and more susceptible to immune clearance^6–8^. Compared to strategies that target viability, anti-virulence strategies may impose less selective pressure for the emergence of resistant strains^2^, and even further diminish the risk of commensal bacteria elimination^9,10^. Considerable works have been conducted to develop anti-virulence strategies, such as the inhibition of expression, secretion, or activity of virulence factors^2,8^.

*Vibrio* species generally inhabit in diverse marine environments. As an emerging cause of bacterial infection, some pathogenic *Vibrio* species infect humans and lead to a variety of clinical symptoms^11,12^. For example, *Vibrio vulnificus* can cause life-threatening septicemia and necrotizing fasciitis with high mortality rates in susceptible individuals^13^. *Vibrio parahaemolyticus* is a leading cause of seafood-borne gastroenteritis worldwide, resulting in diarrhea, nausea, fever, and chills^14^. *Vibrio cholerae*, a causative agent of watery diarrhea, is responsible for large outbreaks of cholera in various countries^15^, and *Vibrio alginolyticus* causes otitis and superficial wound infections in humans^16^. Although many antibiotics such as quinolones and tetracyclines have been applied for the treatment of *Vibrio* infection^11,17^, the recent reports of antibiotic resistant *Vibrios* threaten the efficacies of these antibiotics as treatment options^18,19^. In an effort to develop anti-virulence strategies against pathogenic *Vibrio* species, small molecules targeting virulence of *Vibrio* species have been identified^20–25^. However, very little is known about the molecular mechanisms of the compounds.

HlyU is a conserved transcriptional regulator required for the activation of various virulence genes in *Vibrio* species^14,26–28^. For example, *V. vulnificus* HlyU induces the expression of *vvhA*, *rtxA*, and *plpA* encoding hemolysin, multifunctional-autoprocessing repeats-in-toxin (MARTX) toxin, and phospholipase A_2_, respectively, by directly binding to the promoter region^26,29,30^. Similarly, *V. parahaemolyticus* HlyU directly induces the expression of *exsA*, which is essential for the type III secretion system 1 (T3SS1)^14^. The hemolysin VvhA lyses erythrocytes, damages endothelial cells, and induces inflammatory cell infiltration^31,32^. The MARTX toxin causes host cell rounding by dysregulating actin cytoskeleton and antagonizes phagocytic activity of host immune cells^33–36^. The secretory phospholipase A_2_ PlpA contributes to the lysis and necrotic death of host cells^30^. T3SS1 directly delivers multiple cytopathic and cytotoxic effector proteins into the host cells^37^. Host tissue destruction and inflammation caused by these virulence factors promote the survival, dissemination, and pathogenesis of *V. vulnificus* and *V. parahaemolyticus* in mice^30,38,39^. Accordingly, a deletion mutation of *hlyU* significantly attenuated virulence of the bacteria against human epithelial HeLa cells or mice^14,29^. Therefore, inhibition of the HlyU activity could be a plausible anti-virulence strategy against these *Vibrio* species.

In the present study, we performed high-throughput screening of 8,385 compounds and identified a small-molecule inhibitor of HlyU, CM14, that significantly inhibited the HlyU activity in *V. vulnificus*. CM14 reduced the expression of HlyU-regulated virulence genes, attenuating the virulence-related phenotypes of *V. vulnificus in vitro*, *ex vivo*, and in a mouse model. Biochemical analysis indicated that CM14 prevents HlyU binding to its target promoter DNA. Further mass spectrometric and mutational analyses revealed that a part of CM14 covalently modifies Cys30, a well-conserved residue of HlyU proteins in *Vibrio* species, both *in vitro* and *in vivo*. Remarkably, CM14 decreased the expression of virulence genes and showed anti-virulence effects against other pathogenic *Vibrio* species, without affecting the bacterial growth.

## Results

### Identification of CM14 as an inhibitor of the HlyU activity

**Figure 1:**
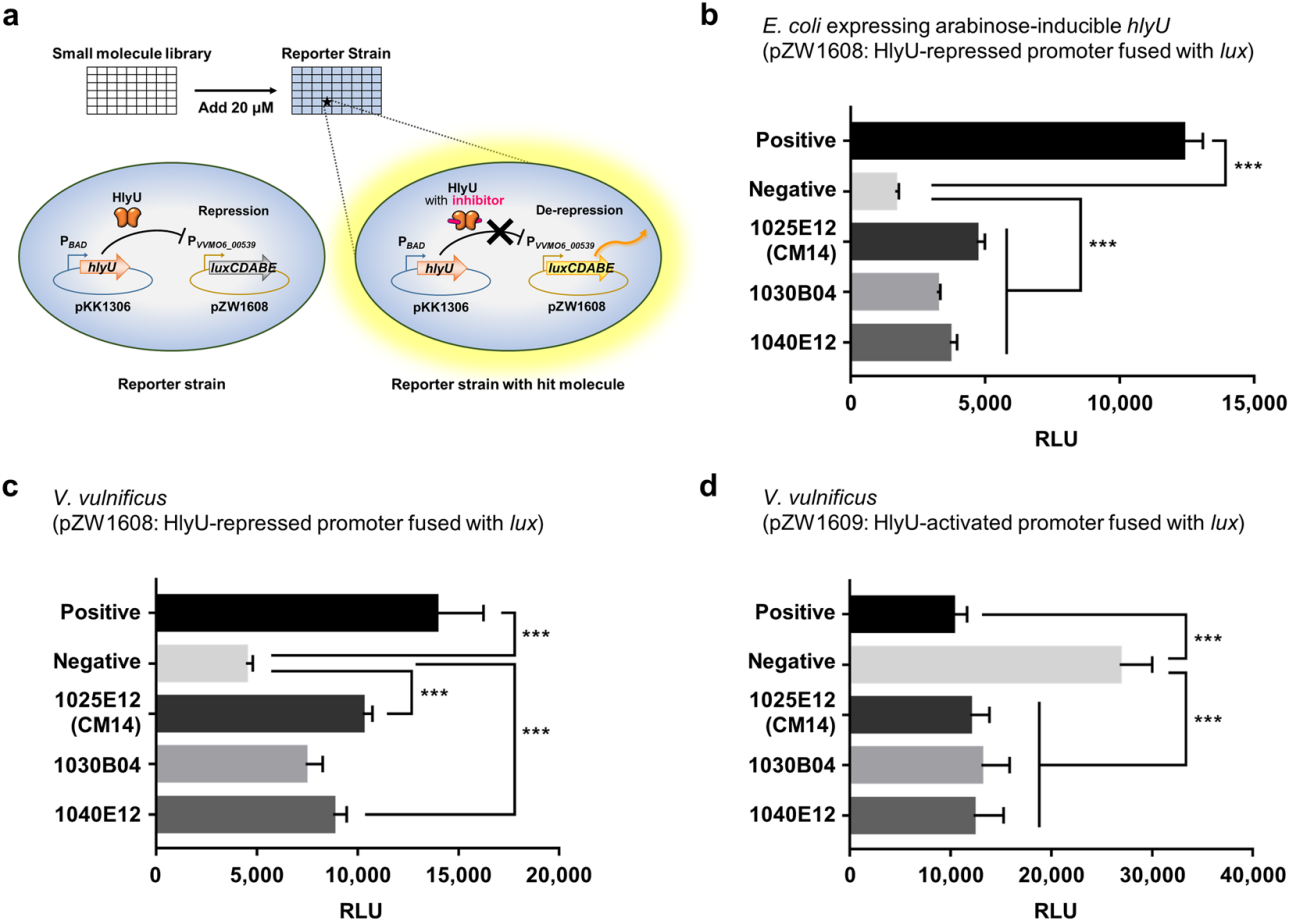
High-throughput screening for HlyU inhibitors. (**a**) Schematic demonstration of high-throughput screening of small molecules. An *E. coli* reporter strain contains pKK1306 expressing HlyU under arabinose-inducible promoter P_*BAD*_ and pZW1608 carrying the *luxCDABE* genes under HlyU-repressed promoter P_*VVMO6_00539*_. (**b**–**d**) Each bar represents RLU of the *E. coli* reporter strain (**b**) and *V. vulnificus* reporter strains containing pZW1608 (**c**) or pZW1609 (**d**) in the presence of hit molecules as indicated. Error bars represent the standard deviation (SD) from biological triplicates. Statistical significance was determined by multiple comparisons after one-way analysis of variance (ANOVA) (***p < 0.0005). 1025E12, 1030B04, and 1040E12, hit molecules; Positive, RLUs from *E. coli* without arabinose (**b**) or *V. vulnificus hlyU* mutant (**c**,**d**); Negative, RLUs from *E. coli* with arabinose (**b**) or *V. vulnificus* wild type (**c**,**d**); RLU, relative luminescence unit.

To identify a specific inhibitor of HlyU, we constructed an *Escherichia coli* reporter strain containing pKK1306 (carrying an arabinose-inducible *hlyU* of *V. vulnificus*) and pZW1608 (carrying a promoterless *lux* operon fused to a promoter P_*VVMO6_00539*_)^40^. Because the VVMO6_00539 gene is directly repressed by HlyU (Fig. 1a; Supplementary Fig. S1a,b), the resulting *E. coli* strain remains non-luminescent in an arabinose-containing media unless a potential hit molecule inhibits either the expression or function of HlyU (Fig. 1a). By using this HlyU-repressed *lux* reporter system instead of the HlyU-activated system, we could eliminate the false identification of luciferase-inhibiting and/or luminescence-absorbing molecules as hits. Due to the lack of a previously discovered ligand or a putative ligand-binding site in HlyU, a random chemical library containing 8,385 small molecules was screened using the *E. coli* reporter strain. From the screening, three hit molecules (1025E12, 1030B04, and 1040E12) were identified as putative HlyU inhibitors (Fig. 1b). These hit molecules were reexamined using the *V. vulnificus* reporter strains containing the same reporter plasmid pZW1608 (Fig. 1c) or pZW1609 (Fig. 1d), respectively. In contrast to pZW1608, pZW1609 carries the promoterless *lux* operon fused to a promoter of the *rtxA* gene, P_*rtxA*_, which is directly induced by HlyU^26^. With each of the hit molecules, the wild-type *V. vulnificus* containing pZW1608 was more luminescent than the negative control (dimethyl sulfoxide, DMSO) (Fig. 1c), while *V. vulnificus* containing pZW1609 was less luminescent than the negative control (Fig. 1d). The use of these two distinct *V. vulnificus* reporter strains verified that the hit inhibitor molecules function directly on HlyU, not on other components such as a luciferase enzyme.

**Figure 2:**
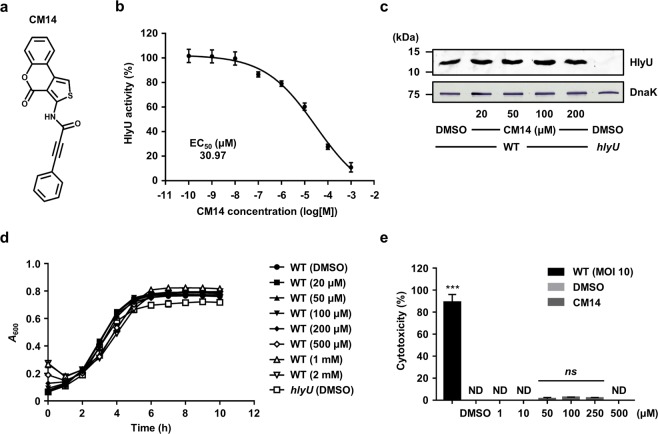
CM14 inhibits the HlyU activity without affecting *V. vulnificus* growth. (**a**) The chemical structure of CM14, *N*-(4-oxo-4H-thieno[3, 4-c]chromen-3-yl)-3-phenylprop-2-ynamide. (**b**) The EC_50_ of CM14 inhibiting HlyU activity was calculated as described in the Methods section from three independent experiments. (**c**) Clear lysate samples of the wild type and *hlyU* mutant cells grown along with various concentrations of CM14 or DMSO (control) were resolved by SDS-PAGE, after which HlyU and DnaK were detected by Western blot analysis. Molecular size markers (Bio-Rad) are shown in kDa. Representative images of Western blot for HlyU and DnaK are shown, and the full-length blots are presented in Supplementary Figure S7. (**d**) Growth of the *V. vulnificus* strains along with various concentrations of CM14 or 2% DMSO (control) was monitored at 1 h intervals using a microplate reader. (**e**) Cytotoxicity was determined using LDH activities released from INT-407 cells incubated at 37 °C for 3 h with various concentrations of CM14, the wild-type *V. vulnificus* (at an MOI of 10), or 2% DMSO (control). The cytotoxicity was expressed using the LDH activity from the cells completely lysed by 5% Triton X-100 as 100%. Error bars represent the SD from the representative of three independent experiments. Statistical significance was determined by one-way ANOVA (**e**) (***p < 0.0005; *ns*, not significant; ND, not detected). WT, wild type; *hlyU*, *hlyU* mutant; MOI, multiplicity of infection.

Among the hit molecules, 1025E12, *N*-(4-oxo-4H-thieno[3,4-c]chromen-3-yl)-3-phenylprop-2-ynamide (C_20_H_11_NO_3_S, molecular weight of 345.37) was most effective in the HlyU inhibition, and thus selected as a HlyU inhibitor and renamed ‘CM14’ (Fig. 2a). The structure of CM14 was confirmed by ^1^H NMR, ^13^C NMR, and mass spectrometric analyses (see Supplementary Information Methods). The HlyU activities were assessed using the wild-type *V. vulnificus* containing pZW1609 in the presence of various concentrations of CM14, and the half maximal effective concentration (EC_50_) of the molecule was determined as 30.97 μM (Fig. 2b). It is noteworthy that CM14 in the range of 20 to 200 μM did not alter the HlyU levels in *V. vulnificus* cells (Fig. 2c), suggesting that CM14 inhibits the activity rather than the cellular levels of HlyU. In addition, CM14 did not affect the growth of *V. vulnificus* (up to 2 mM) and was not toxic to the human epithelial INT-407 cells (up to 500 μM) (Fig. 2d,e). Therefore, these results suggested that CM14 is a small-molecule inhibitor of HlyU activity having a potential to be developed as an anti-virulence agent against *V. vulnificus*.

### CM14 reduces the HlyU-dependent virulence gene expression *in vitro*

**Figure 3:**
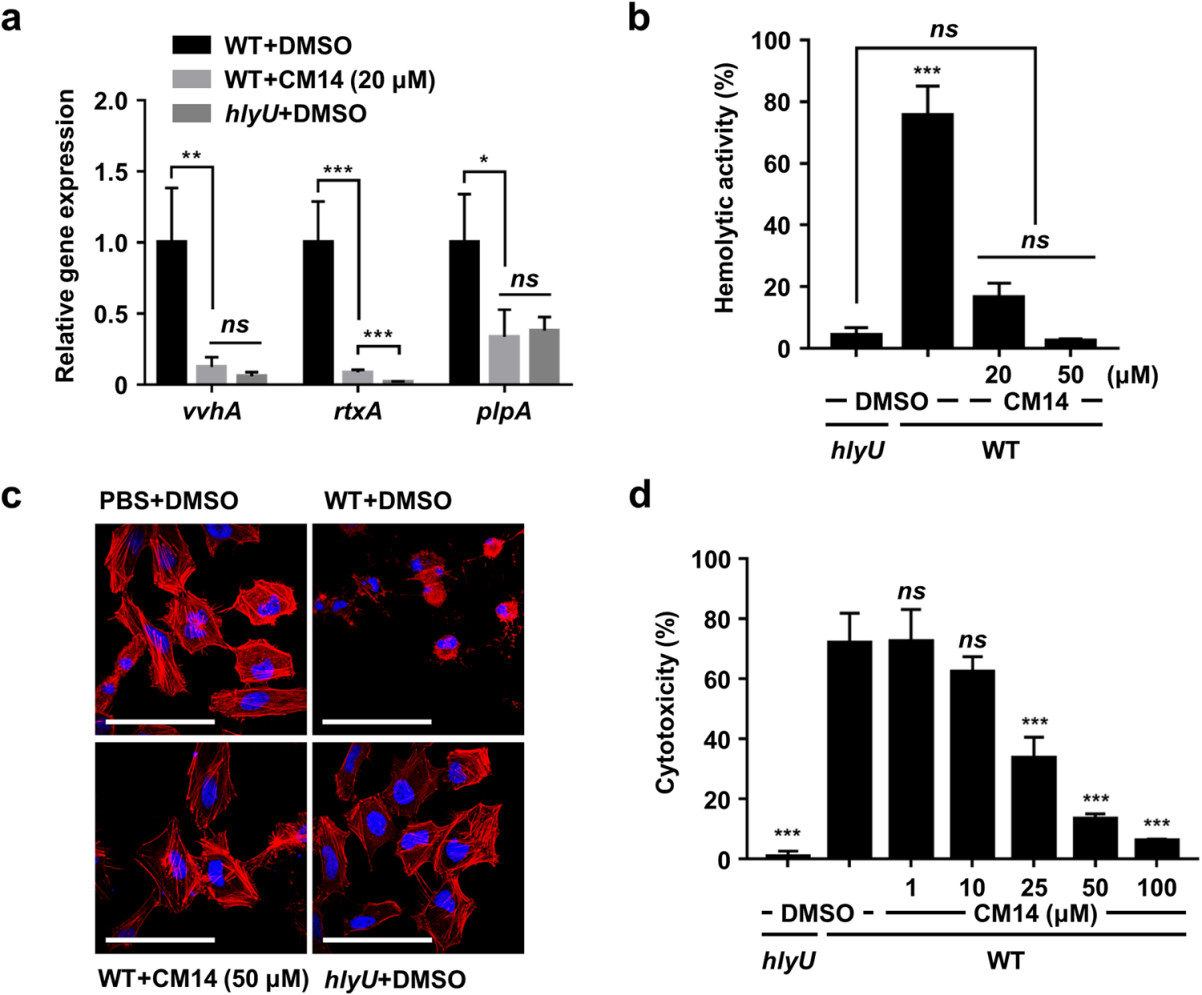
Effects of CM14 on the virulence-related phenotypes of *V. vulnificus*. (**a**,**b**) The *V. vulnificus* strains grown along with CM14 as indicated or DMSO (control) were harvested and fractionated for further analyses. (**a**) The transcript levels of *vvhA*, *rtxA*, and *plpA* in the total RNA of the cells were quantified by qRT-PCR and expressed using each transcript level of the wild type in the presence of DMSO as 1. (**b**) Hemolytic activities of the culture supernatants were determined against human erythrocytes and expressed using complete hemolysis by 5% Triton X-100 as 100%. (**c**) Morphological changes of HeLa cells infected with the *V. vulnificus* strains along with CM14 (50 μM) or DMSO (control) were photographed. Scale bars, 100 μm. (**d**) Cytotoxicity was determined using LDH activities released from INT-407 cells infected with the *V. vulnificus* strains along with CM14 as indicated and expressed using the LDH activity from the cells completely lysed by 5% Triton X-100 as 100%. Error bars represent the SD from three independent experiments (**a**,**b**) and from the representative of three independent experiments (**d**). Statistical significance was determined by the Student’s *t*-test (**a**) and by one-way ANOVA (**b**,**d**) (***p < 0.0005; **p < 0.005; *p < 0.05; *ns*, not significant). WT, wild type; *hlyU*, *hlyU* mutant.

Next, we examined if CM14 affects the expression of *vvhA*, *rtxA*, and *plpA* in *V. vulnificus*. Consistent with the previous result that CM14 inhibits HlyU activity, the transcript levels of *vvhA*, *rtxA*, and *plpA* of the wild-type *V. vulnificus* strain were significantly reduced in the presence of the molecule at 20 μM (Fig. 3a; WT + DMSO vs. WT + CM14). The reduced expression levels of the genes were close to those of the *hlyU* mutant strain ZW141 (Fig. 3a; WT + CM14 vs. *hlyU* + DMSO). We further investigated whether the reduced expression of the virulence genes is reflected in the virulence-related phenotypes. It was reported that *V. vulnificus* VvhA has a hemolytic activity against erythrocytes^31^. Thus, we compared the hemolytic activities in the culture supernatants of the *V. vulnificus* strains grown in the presence or absence of CM14. When incubated with human erythrocytes, the culture supernatant of the wild-type *V. vulnificus* grown in the presence of DMSO control showed robust hemolytic activity (Fig. 3b). In contrast, the culture supernatant of the wild-type *V. vulnificus* grown in the presence of CM14 exhibited significantly reduced (at 20 μM) or nearly no hemolytic activities (at 50 μM) similar to that of the *hlyU* mutant (Fig. 3b). Collectively, these results indicated that the effect of CM14 on the decreased expression of virulence genes is also represented as a reduced virulence-related phenotype of *V. vulnificus in vitro*.

### CM14 attenuates the virulence of *V. vulnificus ex vivo*

The effects of CM14 on the *V. vulnificus*-mediated cytopathic changes of the host cells were assessed *ex vivo*. Since CM14 significantly decreased the *rtxA* transcript level in *V. vulnificus* (Fig. 3a), we first examined whether the molecule prevents the actin cytoskeleton dysregulation primarily caused by the MARTX toxin^35,36^. To this end, we monitored a rapid rounding phenotype of the HeLa cells infected with the *V. vulnificus* strains in the presence or absence of CM14. HeLa cells became round at 1 h post infection of the wild type^41^ (Fig. 3c; WT + DMSO). However, the rounding of HeLa cells was significantly attenuated in the presence of CM14 at 50 μM (Fig. 3c; WT + CM14), and thus the morphology of the cells was comparable to that of the cells with phosphate buffered saline (PBS, vehicle control) or the *hlyU* mutant (Fig. 3c; PBS + DMSO or *hlyU* + DMSO).

Furthermore, the effects of CM14 on the cytotoxicity of *V. vulnificus* were evaluated. For this purpose, lactate dehydrogenase (LDH) release from the INT-407 cells infected with the bacteria was determined. As shown in Fig. 3d, CM14 reduced LDH release from the cells infected with the wild-type *V. vulnificus* in a dose-dependent manner. Notably, 100 μM of CM14 almost abolished the LDH-releasing activity of *V. vulnificus* (Fig. 3d). Taken together, these results revealed that CM14 successfully attenuates the cytopathicity and cytotoxicity of *V. vulnificus ex vivo*.

### CM14 attenuates the pathogenesis of *V. vulnificus* in mice

**Figure 4:**
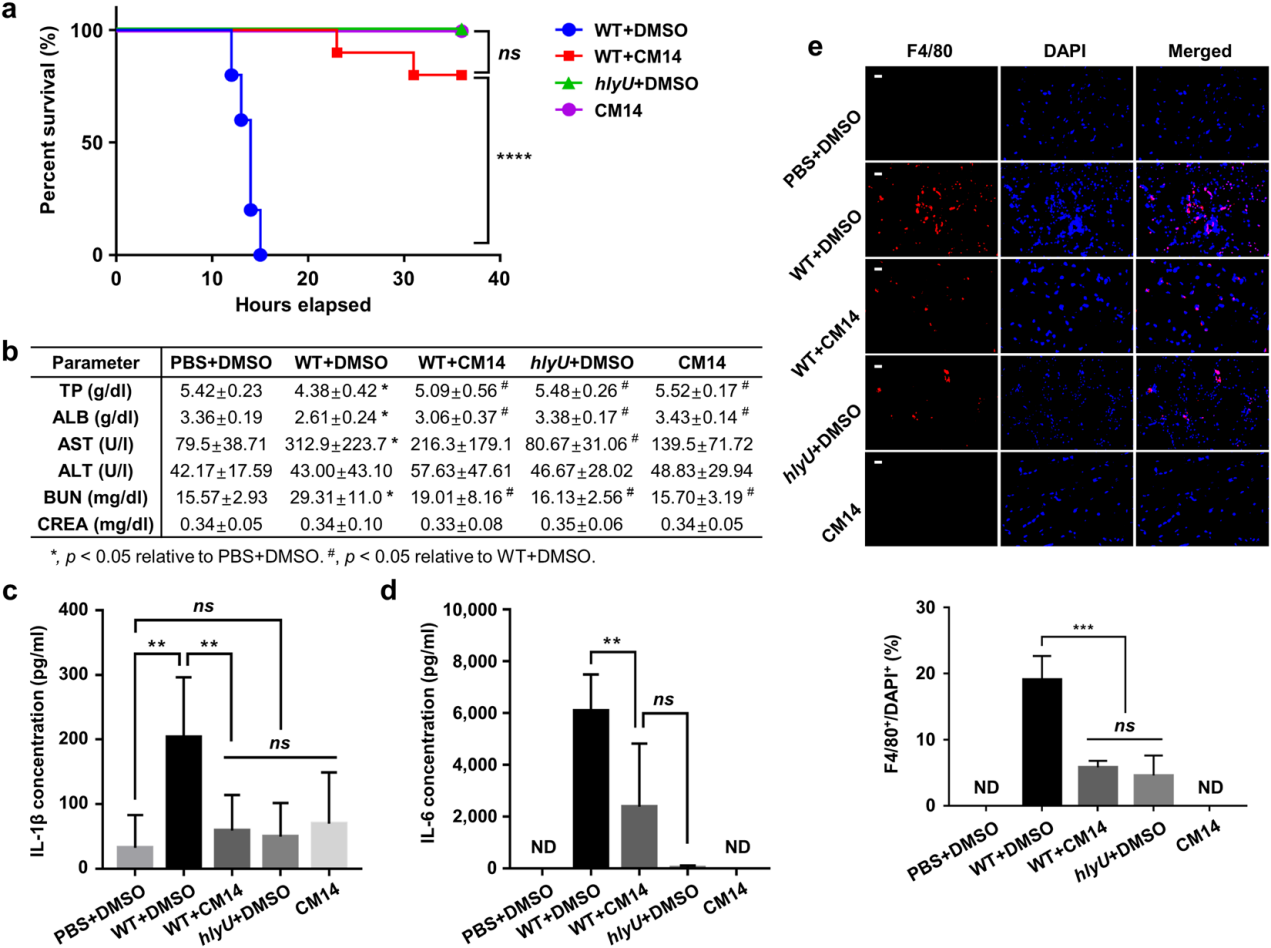
Effects of CM14 on the survival, pathophysiological changes, and inflammatory responses of mice infected with *V. vulnificus*. (**a**) Survival of the mice anesthetized with isoflurane and subcutaneously injected with wild type (*n* = 10), wild type with CM14 (*n* = 10), *hlyU* mutant (*n* = 5) at doses of 7.5 × 10^5^ colony forming unit (CFU), or CM14 alone (*n* = 5, control). (**b**–**e**) The mice, injected as described in (**a**), were sacrificed at 7 h post infection to obtain blood and skin tissue samples. (**b**) The levels of TP, ALB, AST, ALT, BUN, and CREA in the blood plasma of each group [WT + DMSO (*n* = 10), WT + CM14 (*n = *8), *hlyU* + DMSO (*n* = 6), DMSO (*n = *6, control), and CM14 (*n* = 6, control)] were determined by blood biochemical analysis. The data represent the mean ± SD. Statistical significance was determined by multiple comparisons after one-way ANOVA (*p < 0.05 relative to PBS + DMSO; ^#^*p* < 0.05 relative to WT + DMSO). (**c**,**d**) The cytokine levels of IL-1β (**c**) and IL-6 (**d**) in the blood plasma of each group (*n* = 7) were quantified by enzyme-linked immunosorbent assay (ELISA). (**e**) Infiltration of macrophages at the injection sites was determined using skin tissue samples that were immune-stained with F4/80 antibody (for macrophages, red) and DAPI (for nucleus, blue) for counter staining. The percentage of F4/80^+^ cells in DAPI^+^ cells was analyzed by using MetaMorph software. Scale bars, 10 μm (*n* = 4). Error bars represent the SD. Statistical significance was determined by log rank test (**a**) and by multiple comparisons after one-way ANOVA (**c**–**e**) (****p < 0.0001; ***p < 0.0005; **p < 0.005; *p < 0.05; *ns*, not significant; ND, not detected). WT, wild type; *hlyU*, *hlyU* mutant.

To investigate the *in vivo* efficacy of CM14, mortality of mice infected with *V. vulnificus* was evaluated with or without co-administration of the molecule (Fig. 4a). All of the mice infected subcutaneously with the wild type strain were succumbed within 15 h post infection (Fig. 4a; WT + DMSO). In contrast, 80% of the mice survived until the end of experiment (36 h post infection) when CM14 was co-administered at 1.125 mM concentration (1.4 mg/kg body weight) (Fig. 4a; WT + CM14). These results revealed that co-administration of CM14 significantly prolonged the survival of mice infected with *V. vulnificus* (*p* < 0.0001, log rank test). Markedly, the survival rate of the mice infected with the wild type in the presence of CM14 was not statistically different from that of mice infected with the *hlyU* mutant (Fig. 4a; *hlyU* + DMSO). The combined results indicated that CM14 effectively inhibits the pathogenesis of *V. vulnificus* during murine infection.

To examine not only survival but also pathophysiological changes, especially in the degrees of hepatic and renal dysfunction, we analyzed the biochemical parameters in the blood of the mice infected with *V. vulnificus* in the presence or absence of CM14. When mice were infected with the wild type (WT + DMSO), the blood plasma levels of total protein (TP) and albumin (ALB) were decreased, while the levels of aspartate aminotransferase (AST) and blood urea nitrogen (BUN) were increased, compared to the uninfected control mice injected with the vehicle (Fig. 4b; PBS + DMSO). However, the levels of biochemical parameters in mice infected with wild type in the presence of CM14 (WT + CM14) were comparable to those in the control groups such as mice injected with the vehicle or *hlyU* mutant (Fig. 4b; PBS + DMSO or *hlyU* + DMSO). The levels of alanine aminotransferase (ALT) and creatine (CREA) did not show any significant differences among the groups in the conditions tested (Fig. 4b).

Since severe inflammation is accompanied with *V. vulnificus* infection^38,42^, we next assessed immune responses in the *V. vulnificus*-infected mice either co-administered with or without CM14. The pro-inflammatory cytokines interleukin (IL)-1β and IL-6 levels in mouse blood plasma were significantly elevated upon infection of the wild type (Fig. 4c,d; PBS + DMSO vs. WT + DMSO). However, co-administration of CM14 alleviated the secretion of these pro-inflammatory cytokines (Fig. 4c,d; WT + CM14). Consistent with this, the recruitment of F4/80^+^ macrophages to the infection site was also reduced by the administration of CM14 (Fig. 4e). Remarkably, the percentage of F4/80^+^ cells over 4’,6-diamidino-2-phenylindole (DAPI)^+^ cells at the site infected with the wild type in the presence of CM14 was not significantly different from that with the *hlyU* mutant (Fig. 4e). Meanwhile, CM14 did not appear to be toxic to mice, as the levels of blood parameters and macrophage infiltration of the mice injected with CM14 were comparable to those of the mice injected with the vehicle (Fig. 4b to e; CM14 vs. PBS + DMSO). Furthermore, none of the mice injected with CM14 died (Fig. 4a). Taken together, these results indicated that CM14 attenuates the virulence of *V. vulnificus in vivo* and is not toxic toward mice.

### CM14 inhibits the binding of HlyU to its target promoter DNA

**Figure 5:**
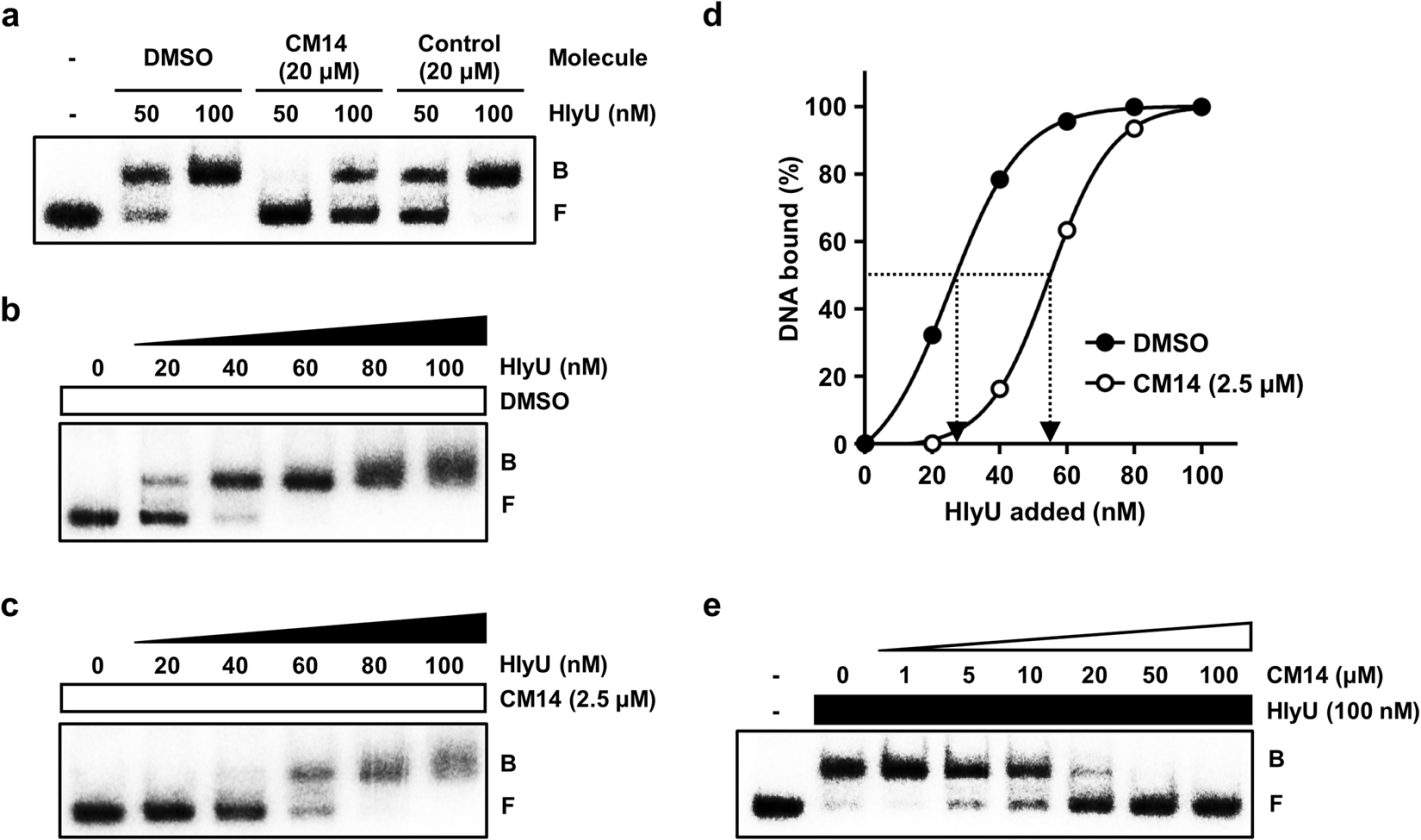
EMSAs of the HlyU and the *rtxA* regulatory DNA complexes. (**a**) HlyU protein (50 or 100 nM) was added to the radioactively-labeled P_*rtxA*_ DNA (5 nM) along with either DMSO (10%), CM14 (20 μM), or a random chemical (20 μM, control), and the complexes were separated by electrophoresis on a 6% nondenaturing polyacrylamide gel. (**b**,**c**) The labeled probe DNA was  mixed with increasing amounts of HlyU as indicated, and then the complexes were separated as described above. (**d**) The relative affinities of HlyU in the presence or absence of CM14 were compared using the data from (**b**,**c**). The concentration of bound DNA was calculated and plotted against the concentration of HlyU added. Each arrow points to the position of half-maximal binding corresponding to the *K*_*d*_. (**e**) HlyU protein (100 nM) was added to the labeled probe DNA along with increasing concentrations of CM14 as indicated, and then the complexes were separated as described above. The cropped gel images are shown, and the full-length gels are presented in Supplementary Figure S8a to d. B, bound DNA; F, free DNA.

As a transcriptional regulator, HlyU functions by binding directly to its target DNA^14,26,30,43^. Thus, we examined whether CM14 inhibits the activity of HlyU by altering the DNA binding of HlyU. Electrophoretic mobility shift assays (EMSAs) revealed that HlyU bound to the target P_*rtxA*_ DNA and resulted in a retarded band of the DNA-HlyU complex in a HlyU concentration-dependent manner (Fig. 5a, DMSO). When 20 μM of CM14 was added, however, the HlyU binding to the DNA decreased, as less amount of retarded bands were detected compared to the DMSO-added control (Fig. 5a; CM14). In contrast, a random molecule that showed no HlyU-inhibiting activity in the screening did not affect HlyU binding to the DNA (Fig. 5a; Control). To determine the effect of CM14 on the dissociation constant (*K*_*d*_) for HlyU, additional EMSA experiments were performed (Fig. 5b,c). Based on the concentration of HlyU required to bind 50% of the DNA probe, the *K*_*d*_ for HlyU without CM14 was estimated as 25.16 nM, while that with 2.5 µM of CM14 was estimated as 54.83 nM (Fig. 5d), indicating that the molecule significantly affects the equilibrium between free and DNA-bound HlyU proteins in the binding reaction. Indeed, the addition of increasing amounts of CM14 resulted in a concentration-dependent inhibition of HlyU binding to the DNA, and 50 μM of CM14 completely abolished the formation of the DNA-HlyU complex (Fig. 5e). Together, the results suggested that inhibition of HlyU binding to its target DNA is a possible mechanism of CM14.

### Chemical modification of HlyU by CM14

**Figure 6:**
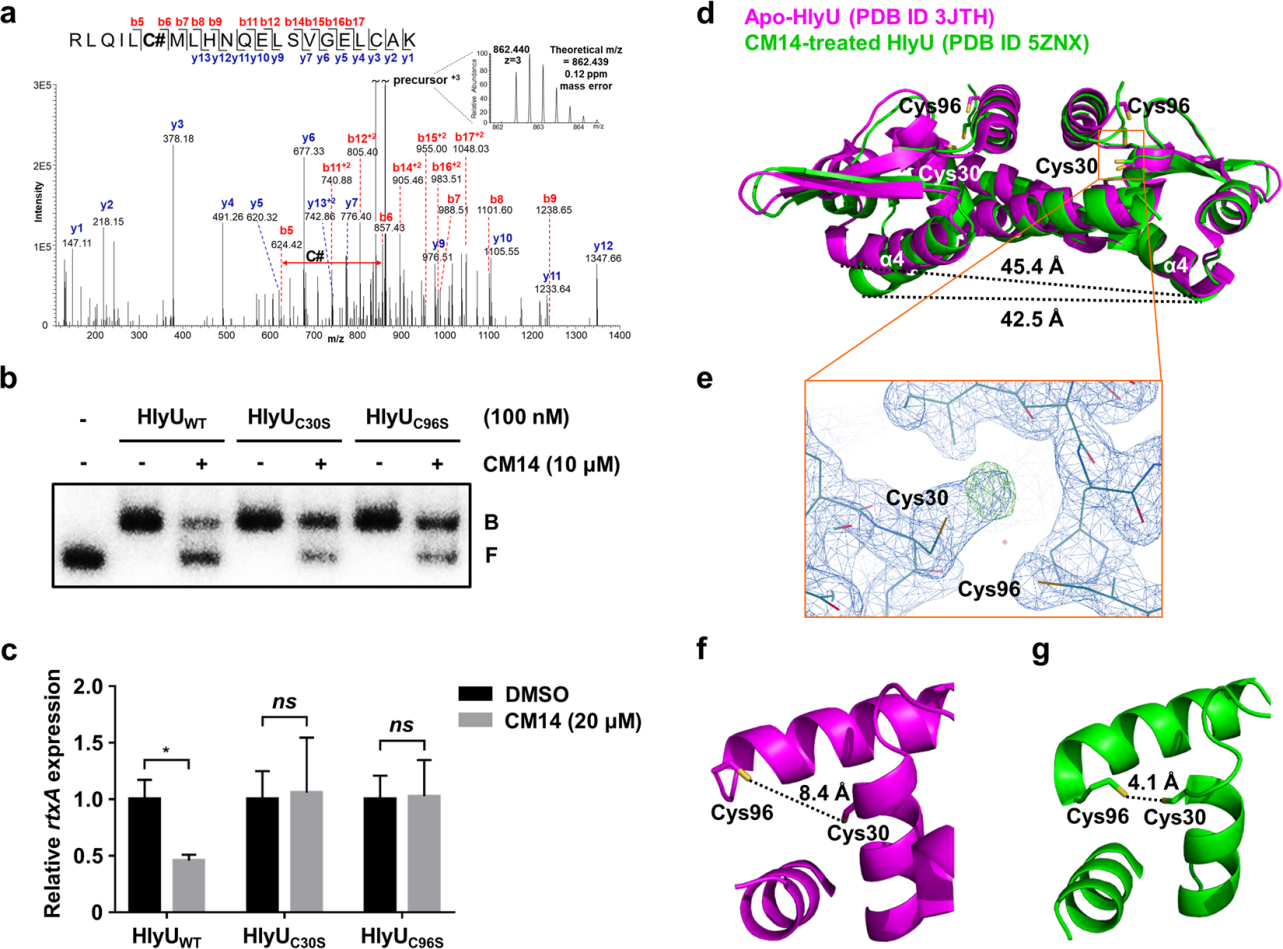
Chemical modification of the Cys30 residue of HlyU by CM14. (**a**) MS/MS spectrum of Cys30-modified peptide (RLQIL**C#**MLHNQELSVGELCAK) from the CM14-treated HlyU protein. C# indicates the mass shift of C_9_H_6_O (# = +130.042 Da) by the cysteine modification. Both N- and C-terminal fragment ion series are represented as b and y series, respectively (e.g. b5, b6, b7… and y1, y2, y3…), and the annotated fragment ions are marked in the inserted peptide sequence. The observed precursor ion (monoisotopic m/z 862.440) in the inserted high resolution MS spectrum matched exactly with a theoretical m/z (862.439). (**b**) EMSA of the wild-type (WT), C30S mutant, or C96S mutant HlyU proteins and the radioactively-labeled P_*rtxA*_ DNA complexes. The HlyU proteins (100 nM) were added to the probe DNA (5 nM) along with either 10% DMSO (control) or CM14 (10 μM) as indicated, and then the complexes were separated as described in Fig. 5. The cropped gel image is shown, and the full-length gel is presented in Supplementary Figure S8e. B, bound DNA; F, free DNA. (**c**) The *hlyU* mutants containing plasmids expressing wild-type or mutant HlyUs as indicated were grown along with CM14 (20 μM) or DMSO (control). The *rtxA* transcript levels in the total RNA of the cells were quantified by qRT-PCR and expressed using the transcript level of each group in the presence of DMSO as 1. Error bars represent the SD from biological triplicates. Statistical significance was determined by the Student’s *t*-test (*p < 0.05; *ns*, not significant). (**d**) Structural comparison of the CM14-treated HlyU (green, PDB code: 5ZNX) and the apo-HlyU (magenta, PDB code: 3JTH). The distances between the DNA-binding helices (𝛼4) are indicated. (**e**) Electron density map around Cys30 of the CM14-treated HlyU structure. The 2*F*_*o*_–*F*_*c*_ (blue mesh) and the *F*_*o*_–*F*_*c*_ (green mesh) maps are contoured at 1.5 σ and 4.4 σ, respectively. (**f**,**g**) Close-up views around Cys30 and Cys96 of the apo-HlyU (**f**) and the CM14-treated HlyU (**g**). The distances between sulfur atoms of the two cysteine residues are indicated.

The possible mechanism of CM14 to inhibit the DNA-binding activity of HlyU was further investigated at a molecular level. To this end, tandem mass spectrometric analysis was performed for the CM14-treated HlyU sample. Figure 6a clearly showed that the Cys30 residue (C#) in the HlyU peptide, RLQILC#MLHNQELSVGELCAK, was covalently modified by the moiety with molecular mass of 130.042 Da, indicating that a certain part of CM14, probably consisting of C_9_H_7_O, is attached to the Cys30 of HlyU. Importantly, this modification seems to occur *in vivo* as well, because the freshly purified HlyU protein from the CM14 (50 μM)-treated *E. coli* cells also revealed the same result (Supplementary Fig. S2a). To verify this modification on the Cys30, a mutant HlyU protein with Cys to Ser substitution at Cys30 (HlyU_C30S_) was prepared and reacted with CM14. When the resulting mixture was analyzed by tandem mass spectrometry, a spectrum corresponding to the HlyU peptide containing a substituted serine, but not containing the covalently modified moiety, was detected (Supplementary Fig. S2b), indicating that the thiol group of Cys30 is important for the covalent modification. Consistent with this, the mutant HlyU_C30S_ became resistant to CM14, as supported by the observations that the DNA-binding activity of HlyU_C30S_ was less affected by the molecule *in vitro* (Fig. 6b) and that the expression of *rtxA* was not attenuated by the molecule *in vivo* (Fig. 6c).

According to the previously determined crystal structure of HlyU, there is another Cys residue, Cys96, near the Cys30 (Supplementary Fig. S2c, PDB code: 3JTH). To examine the role of Cys96 on the CM14-mediated modification of Cys30, this residue was also substituted with Ser. The resulting HlyU_C96S_ was also resistant to CM14 *in vitro* and *in vivo*, as was HlyU_C30S_ (Fig. 6b,c; HlyU_C96S_). Notably, however, Cys96 residue was detected unmodified in the above tandem mass spectrometric analysis of CM14-treated HlyU sample. Taken together, the results indicated that CM14 reacts with the thiol group of Cys30 of HlyU via a putative chemical reaction involving Cys96, and consequently inhibits the DNA-binding activity of HlyU.

To gain insights into the structural influence of CM14 on HlyU, we determined the crystal structure of CM14-treated HlyU protein at 2.1 Å resolution and compared it with the previously determined apo-HlyU structure^44^ (PDB code: 3JTH) (Fig. 6d,e). The overall structure of the CM14-treated HlyU is similar to that of apo-HlyU (Fig. 6d). However, there is an extra electron density map around Cys30 of the CM14-treated HlyU suggesting a potential chemical modification of Cys30 (Fig. 6e). Although the moiety attached to Cys30 is partially visible presumably due to the high flexibility, this observation is consistent with the above result that CM14 modifies the Cys30 of HlyU (Fig. 6a). Notably, further comparison revealed that CM14 induces a conformational change of HlyU, thereby substantially decreasing the distance between Cys30 and Cys96 from 8.4 Å to 4.1 Å (Fig. 6f,g). In addition, we found that the distance between two DNA-binding 𝛼-helices (𝛼4) in HlyU dimer by 2.9 Å (Fig. 6d), which may account for the impaired DNA-binding activity of HlyU (Fig. 5).

### CM14 exhibits anti-virulence effects against other *Vibrio* species

**Figure 7:**
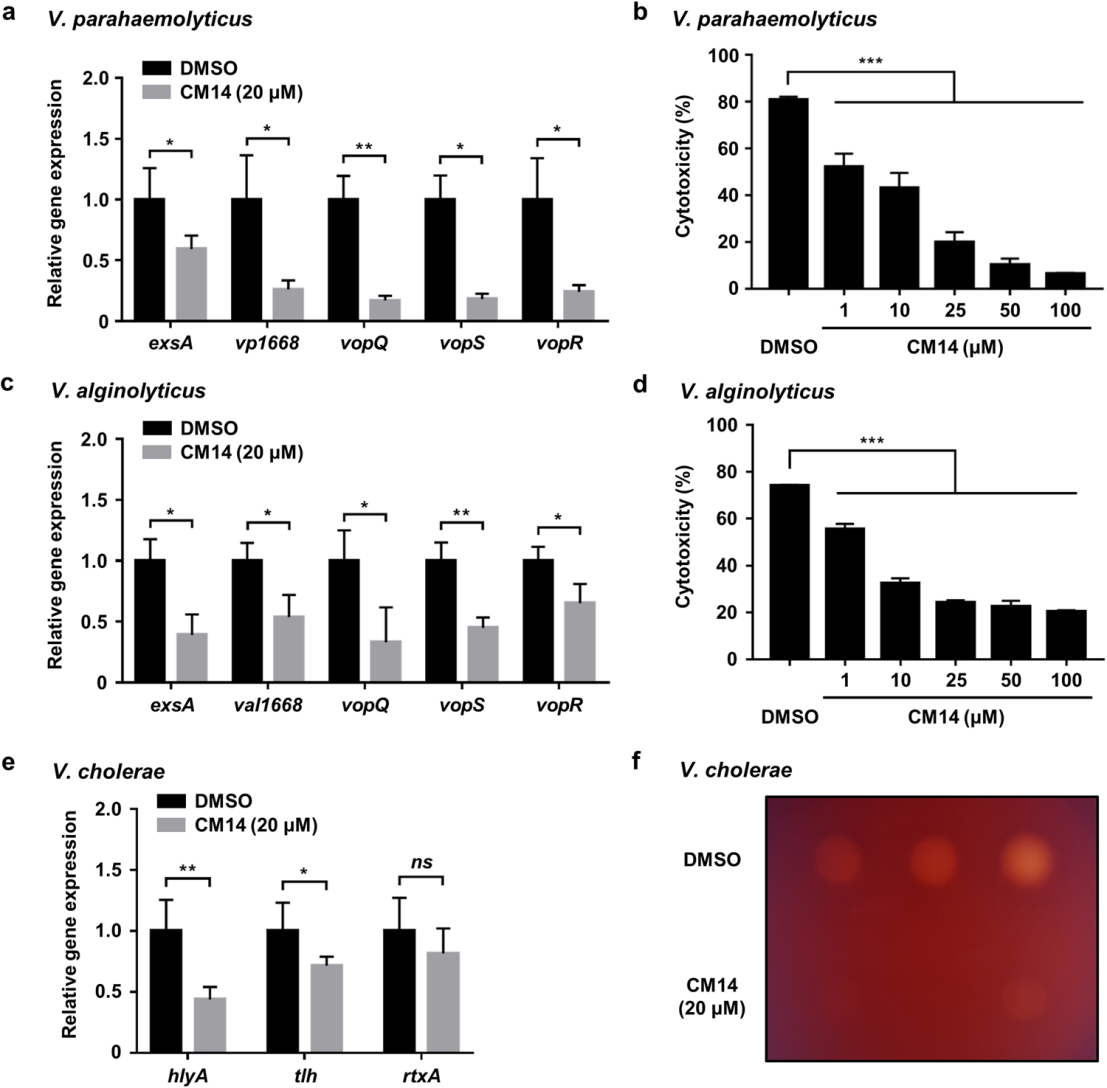
CM14 is effective in attenuating virulence of other *Vibrio* species. (**a**) *V. parahaemolyticus* was grown in T3SS1 inducing condition for 3 h along with CM14 (20 μM) or DMSO (control). The transcript levels of *exsA*, *vp1668*, *vopQ*, *vopS*, and *vopR* in the total RNA of the cells were quantified by qRT-PCR and expressed using each transcript level in the presence of DMSO as 1. (**b**) Cytotoxicity was determined using LDH activities released from INT-407 cells infected with *V. parahaemolyticus* at an MOI of 10 along with CM14 as indicated for 2 h and expressed using the LDH activity from the cells completely lysed by 5% Triton X-100 as 100%. (**c**) *V. alginolyticus* was grown to *A*_600_ of 0.5 along with CM14 (20 μM) or 2% DMSO (control). The transcript levels of each gene in the total RNA of the cells were quantified by qRT-PCR and expressed using each transcript level in the presence of DMSO as 1. (**d**) Cytotoxicity of *V. alginolyticus* was determined as described in (**b**), except that *V. alginolyticus* infection was performed for 4 h. (**e**,**f**) *V. cholerae* grown to *A*_600_ of 0.5 along with CM14 (20 μM) or 2% DMSO (control) were harvested and fractionated for further analyses. (**e**) The transcript levels of *hlyA*, *tlh*, and *rtxA* in the total RNA of the cells were quantified by qRT-PCR and expressed using each transcript level in the presence of DMSO as 1. (**f**) Hemolytic activities of the culture supernatants of *V. cholerae*. Ten microliters of the concentrated supernatants were spotted onto 7% horse blood agar plate. Three different culture supernatants were spotted and monitored after incubation at 37 °C for 24 h. Error bars represent the SD from more than three biological replicates (**a**,**c**,**e**) and from the representative of three independent experiments (**b**,**d**). Statistical significance was determined by the Student’s *t*-test (**a**,**c**,**e**) and by one-way ANOVA (**b**,**d**) (***p < 0.0005; **p < 0.005; *p < 0.05; *ns*, not significant).

HlyU proteins are highly conserved in *Vibrio* species and show high degree of sequence similarity. Especially, the residues Cys30 and Cys96 are well conserved in HlyU homologues of common pathogenic *Vibrios*, including *V. parahaemolyticus*, *V. alginolyticus*, and *V. cholerae*^43^ (Supplementary Fig. S3). Thus, we hypothesized that CM14 would be effective against other *Vibrio* species harboring HlyU homologue. Unfortunately, the homologues of *rtxA* and *vvhA* are absent in *V. parahaemolyticus* and *V. alginolyticus*, while the *plpA* homologue is present. However, the *plpA* homologues have not been reported to be regulated by HlyU. Accordingly, we examined the expression of *exsA* in *V. parahaemolyticus* which is directly induced by HlyU^14^. As expected, CM14 significantly reduced the *exsA* expression in *V. parahaemolyticus* (Fig. 7a). Since ExsA positively regulates multiple T3SS1-associated genes^14^, we further examined the expression of T3SS1 genes^37,45^ in the presence or absence of CM14. Again, the expression of tested T3SS1 genes, such as *vp1668, vopQ, vopS*, and *vopR* was significantly attenuated by CM14 treatment (Fig. 7a). Moreover, this molecule reduced the cytotoxicity of *V. parahaemolyticus* against the INT-407 cells in a dose-dependent manner (Fig. 7b).

Next, the effects of CM14 on *V. alginolyticus* and *V. cholerae* were examined. Since *V. alginolyticus* possesses T3SS which is particularly similar to that of *V. parahaemolyticus*^16^, we assumed that HlyU may also regulate T3SS in *V. alginolyticus*. In *V. cholerae*, HlyU activates the expression of *hlyA* by directly binding to the promoter region^43^. As shown in Fig. 7c to f, CM14 markedly inhibited the expression of *exsA* and T3SS genes (*val1668*, *vopQ*, *vopS*, and *vopR*) in *V. alginolyticus* and two divergently transcribed hemolysin genes (*hlyA* and *tlh*) in *V. cholerae*, thereby attenuating cytotoxicity or hemolytic activity of the *Vibrios*. Notably, CM14 did not hamper the growth of *V. parahaemolyticus*, *V. alginolyticus*, and *V. cholerae* (Supplementary Fig. S4), as in the case of *V. vulnificus* (Fig. 2d).

## Discussion

Numerous bacterial genes encoding virulence factors required for overall success in the pathogenesis have been identified^46,47^. Many of these genes are coordinately regulated by a common global regulatory protein(s) to obtain their effective cooperation during infection^48,49^. Therefore, inhibiting the activity of global regulatory proteins is a promising strategy that can prevent the production of virulence factors simultaneously and thereby impede bacterial pathogenesis efficiently^1,2,6^. HlyU homologue in *Vibrio* species is a key regulatory protein that induces the expression of various virulence genes, suggesting that it could be an attractive target to develop the anti-virulence strategies against the pathogenic *Vibrios*. In the present study, we have identified and characterized a small molecule, CM14, that specifically inhibits HlyU activity, thus attenuating the pathogenesis of *V. vulnificus* without suppressing its growth. As expected, it also attenuated virulence phenotypes of other pathogenic *Vibrios*.

Among the genes regulated by HlyU in *V. vulnificus*, the expressions of VVMO6_00539 and VVMO6_03281 which are directly repressed by the protein (Supplementary Fig. S1a to c) were significantly induced in the presence of CM14 (Supplementary Fig. S1d,e). These results indicated that CM14 inhibits HlyU activity regardless of its regulatory mode, and also suggested that the molecule functions at a stage of HlyU binding to the target promoter DNA rather than other stages such as interaction of the protein with RNA polymerase. Indeed, the EMSA results revealed that CM14 directly inhibits DNA-HlyU interaction (Fig. 5). This inhibitory mode of action is advantageous in controlling pathogenesis of the bacteria because it blocks the production of virulence factors at the earliest step^2,5^.

To the best of our knowledge, CM14 is the first compound that covalently modifies HlyU and inhibits the virulence of *V. vulnificus* in a mammalian infection model. Although two compounds, fursultiamine hydrochloride and 2′,4′-dihydroxychalcone, have been identified as HlyU inhibitors, their mode of action was barely demonstrated^24,25^. Moreover, both of them failed to show *in vivo* efficacy in an animal model, and the latter even impeded bacterial growth at the low concentration of 15 μM. From the structural point of view, compared to the two compounds, CM14 is endowed with a novel keto-alkyne moiety that is required for the covalent modification of Cys30 in HlyU (see below).

Acute failures of liver and kidney in *V. vulnificus*-infected patients are the key pathophysiological features associated with fatal death^50,51^. Our study revealed that the inhibition of HlyU activity by CM14 suppressed the hepatic and renal dysfunction (Fig. 4b) and subsequently increased the survival rate of mice infected with *V. vulnificus* (Fig. 4a). In addition, our data showed that CM14 reduces both the production of pro-inflammatory cytokines in the blood plasma (Fig. 4c,d) and the massive recruitment of macrophages to the infection site (Fig. 4e). Because the MARTX toxin and VvhA induce pro-inflammatory cytokine production in mice^38^ and these cytokines trigger the recruitment of immune cells such as macrophages^52,53^, the *in vivo* results indicate that CM14 alleviates the clinical manifestations related to the *V. vulnificus*-induced septicemia by down-regulating the virulence factors. Since these virulence factors are also crucial for the invading pathogen to combat against residing immune cells and thus to proliferate/disseminate in the host^30,33,38^, *V. vulnificus* cells attenuated by the molecule might be readily cleared out of the mice.

**Figure 8:**
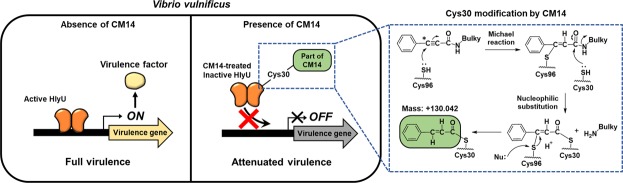
Proposed molecular mechanism underlying the CM14-mediated inhibition of HlyU binding to target DNA. A possible reaction mechanism for the Cys30 modification of HlyU by CM14 is shown in a blue dashed box on the right. First, the sulfur atom of Cys96 of HlyU reacts with a carbon atom (asterisk) of CM14, a Michael reaction acceptor site. Then, a sulfur atom of Cys30 of HlyU attacks a carbonyl carbon of CM14 and releases an amine group with bulky rings. Subsequently, a nucleophile (Nu, e.g. His92) around the reaction site cleaves the sulfur-carbon bond between Cys96 and the remaining part of CM14, protonating the carbon to create the carbon-carbon double bond. The remaining part of CM14 (represented by a green rounded box) is covalently linked to the sulfur atom of Cys30. Active HlyU can bind to target promoter DNA, leading to the production of virulence factors and making *V. vulnificus* fully virulent. In contrast, inactivation of HlyU by CM14 inhibits the DNA binding of HlyU, resulting in reduced expression of HlyU-regulated virulence genes. These events eventually attenuate the virulence of *V. vulnificus*.

Given the clear mass spectrometric evidence and biochemical data (Fig. 6a to c), we concluded that the Cys30 residue of HlyU was covalently modified with a certain part of CM14 consisting of C_9_H_7_O, and the Cys96 residue participated in this modification reaction. Based on these observations, we propose a possible chemical reaction mechanism for the covalent modification of HlyU by CM14 (Fig. 8; see the blue dashed box on the right). In the proposed reaction, the sulfur atom of Cys96 may first attack a carbon atom of the carbon-carbon triple bond of CM14. The second attack by the sulfur atom of Cys30 would release the amine group with the bulky rings, remaining a part with the phenyl group of CM14. Subsequently, a nucleophile (e.g. His92; Supplementary Fig. S2c) around the reaction site would cleave the sulfur-carbon bond between Cys96 and the remaining part of CM14, and the carbon is protonated, leaving the C_9_H_7_O moiety on Cys30.

Notably, CM14 seems specific for HlyU among various thiol-dependent transcriptional regulators, because only the HlyU regulon was differentially regulated by CM14 in the whole transcriptome sequencing analysis (Supplementary Fig. S5a). Indeed, samples of WT + CM14, *hlyU* + DMSO, *hlyU* + CM14 were clustered into a certain group that is distinct from the WT + DMSO samples in a principal component analysis (Supplementary Fig. S5b). We thus hypothesize that the bulky rings of CM14 may be involved in the specific interaction with HlyU at the early steps of binding, but the details of interactions including binding constant remain to be studied in the future. The effects of CM14 on thiol groups of other proteins such as those in the host should also be clarified by future studies.

Nonetheless, how does this modification affect the DNA-binding activity of HlyU protein? Intriguingly, a previous simulation study on the *V. cholerae* HlyU protein revealed that a distance between Cys38 and Cys104, which correspond to the Cys30 and Cys96 of *V. vulnificus* HlyU, respectively, has a correlation with the target DNA binding. Specifically, the distance between Cys38 and Cys104 is 8.67 Å when the protein is expected to bind to a target DNA^43^. From the comparison of the crystal structure of CM14-treated HlyU with that of apo-HlyU (Fig. 6d), we found that the distance between Cys30 and Cys96 residues was significantly shortened from 8.4 Å to 4.1 Å upon CM14 treatment (Fig. 6f,g). Furthermore, the distance between two DNA-binding 𝛼-helices (𝛼4) in HlyU dimer was also decreased by 2.9 Å (Fig. 6d). Altogether, the results indicate that CM14-mediated Cys30 modification substantially changes the HlyU conformation, and thus inhibits HlyU binding to target DNA (Figs 5 and 8).

Increasing number of studies have reported small molecules that can inhibit the activity or expression of virulence factors without affecting bacterial growth. For instance, Virstatin precludes dimerization of *V. cholerae* ToxT and prevents the expression of cholera toxin and toxin coregulated pilus^54,55^. Similarly, LED209 inhibits QseC activity, reducing the QseC-dependent virulence gene expression and virulence of multiple Gram-negative pathogens^56,57^. ITC-12 covalently modifies a cysteine residue of LasR, inhibits quorum sensing-mediated gene expression, and attenuates virulence of *Pseudomonas aeruginosa*^58^. Ebselen binds to an active cysteine residue in the cysteine protease domain and thereby inhibits the autoproteolytic cleavage of TcdA and TcdB, the *Clostridium difficile* major toxins^59^. Interestingly, CM14, in addition to ITC-12 and Ebselen, also covalently modifies Cys30 of *V. vulnificus* HlyU (Fig. 6a), supporting the present idea that cysteine residues, along with their scarcity and enhanced reactivity, can be good targets for the development of selective inhibitors of proteins^60^.

CM14 successfully inhibited the expression of various virulence genes in *Vibrio* species, including *vvhA*, *rtxA*, and *plpA* of *V. vulnificus* (Fig. 3a), T3SS1 genes of *V. parahaemolyticus* (Fig. 7a), T3SS genes of *V. alginolyticus* (Fig. 7c), and *hlyA* and *tlh* of *V. cholerae* (Fig. 7e). Consistent with the previous report that the promoter region of *rtxA* in *V. cholerae* is not directly bound by the HlyU protein^61^, the expression of *rtxA* in *V. cholera*e was not affected by CM14 (Fig. 7e). This is noteworthy because it further supports that CM14 specifically affects the HlyU protein. Nevertheless, these results suggest that CM14 has a broad-spectrum anti-virulence effect against pathogenic *Vibrio* species harboring HlyU homologue to regulate the expression of diverse virulence genes.

In conclusion, we identified a small molecule CM14 which inhibits HlyU activity by covalently modifying Cys30 and thus attenuates the virulence of *Vibrio* species. CM14 exhibited its anti-virulence effect even at the post-infection treatment, although it was *ex vivo* case (Supplementary Fig. S6). Further studies are needed to explore the potential of CM14 as a therapeutic agent against *V. vulnificus* infection, including the evaluation of CM14 analogues with improved bioavailability. Since CM14 does not hamper the bacterial growth, it would present no or low selective pressure for the development of resistance.

## Methods

### Strains, plasmids, culture conditions, and high-throughput screening

The strains and plasmids used in this study are listed in Supplementary Table S1. *E. coli* and *V. vulnificus* strains were grown in Luria-Bertani (LB) medium and LB supplemented with 2% (w/v) NaCl (LBS) at 37 °C and 30 °C, respectively. *V. parahaemolyticus*, *V. alginolyticus*, and *V. cholerae* were grown in LBS, tryptic soy broth supplemented with 1% (w/v) NaCl, and LB, respectively, at 37 °C. For T3SS1 inducing condition, *V. parahaemolyticus* was grown in Dulbecco’s modified Eagle’s medium (DMEM) supplemented with 1% fetal bovine serum (FBS)^45^. Bacterial growth was monitored spectrophotometrically at 600 nm (*A*_600_). HeLa cells originated from the American Type Culture Collection were maintained at 37 °C with 5% CO_2_ in DMEM containing 10% FBS, 50 μg/ml penicillin and 50 μg/ml streptomycin. For infection experiments, the cells were washed with pre-warmed PBS and kept in fresh DMEM. The small molecule libraries (each dissolved in 100% DMSO at 1 mM) were kindly provided by the Korea Chemical Bank (http://www.chembank.org). Detailed descriptions on construction of the *E. coli* reporter strain, information related to the high-throughput screening and results are provided in Supplementary Information Methods and Supplementary Table S2.

### Determination of the EC_50_ of CM14

To determine EC_50_ (the concentration of CM14 inhibiting the HlyU activity by 50%), the wild-type *V. vulnificus* reporter strain containing pZW1609 (Supplementary Table S1), a HlyU-activated reporter plasmid, was exposed to various concentrations (10^−10^ to 10^−3^ M) of CM14. Luminescence and growth (*A*_600_) of the reporter strain were measured after 1.5 h incubation using a microplate reader (Infinite™ M200 microplate reader, Tecan, Männedorf, Switzerland), and RLUs were calculated by dividing luminescence with *A*_600_^62^. The HlyU activities were expressed using the RLU observed in the absence of CM14 (in the presence of 2% DMSO) as 100%. The EC_50_ was calculated by plotting the relative HlyU activities versus the CM14 concentrations using GraphPad Prism 7.0 (GraphPad Software, San Diego, CA).

### Western blot and transcript analyses

The *V. vulnificus* strains along with CM14 or 2% DMSO were grown to *A*_600_ of 0.5 and used to analyze either the HlyU protein or the *vvhA*, *rtxA*, and *plpA* transcript levels. HlyU and DnaK in the cell lysates were detected using rabbit anti-*V. vulnificus* HlyU antibody and mouse anti-*E. coli* DnaK antibody (Enzo lifescience, Farmingdale, NY) by Western blot analysis. Expression of specific genes was determined by qRT-PCR with a pair of specific primers (Supplementary Table S3). Relative expression levels of each gene were calculated by using the 16S rRNA expression level as the internal reference for normalization.

### Virulence assays

To determine hemolytic activity *in vitro*, the *V. vulnificus* strains grown to *A*_600_ of 1.0 along with CM14 or 2% DMSO (control) were harvested and fractionated into cells and supernatants by centrifugation. The culture supernatants were purified through Puradisc^TM^ 25 mm syringe filter (pore size 0.2 μm; GE healthcare, Menlo Park, CA) and concentrated using Amicon Ultra-15 (cut-off 10 kDa; Millipore, Temecula, CA). An aliquot of the supernatants was mixed with an equal volume of human erythrocytes (10% in PBS; Innovative Research, Novi, MI) and incubated at 37 °C for 3 h. The hemolytic activity was measured by spectrophotometry as described previously^63^.

Two different assays were performed to determine cytopathicity and cytotoxicity of the *V. vulnificus* strains *ex vivo*. To examine the cytopathic changes, HeLa cells grown in a µ-slide 4-well plates (Ibidi, Germany) were infected with the *V. vulnificus* strains at an MOI of 2 along with 50 μM of CM14 or 1% DMSO (control). After 1 h incubation at 37 °C, the cells were washed and fixed, and nuclei and actin of the cells were stained with Hoechst® 33342 (final 5 μg/ml; Thermo Fisher Scientific, Waltham, MA) and with rhodamine-phalloidin (one unit per microscope slide; Thermo Fisher Scientific), respectively. Cell morphological changes were photographed using a laser scanning confocal microscope (C2plus, Nikon, Japan) and analyzed using NIS-Elements software (Nikon). To examine cytotoxicity, the monolayers of INT-407 cells (HeLa cell-derived epithelial cells) grown in a 96-well tissue culture plate (Nunc, Roskilde, Denmark) were infected with *V. vulnificus* strains at an MOI of 10 along with various concentrations of CM14 or 1% DMSO (control). After 2.5 h incubation at 37 °C, the LDH activities in the supernatant were measured as described previously^30^.

### Mouse infection assays

All manipulations for mouse infection assay were performed following the National Institutes of Health Guidelines for Humane Treatment and approved by the Animal Care and Use Committee of Seoul National University (SNU-170417-26-2). Mouse mortality, blood biochemical parameters, pro-inflammatory cytokine production, and macrophage infiltration were evaluated to determine the virulence of *V. vulnificus in vivo*. For the mouse mortality test, the *V. vulnificus* strains grown to *A*_600_ of 0.5 were harvested and suspended in PBS to 7.5 × 10^6^ CFU/ml. Groups of Institute of Cancer Research (ICR) female mice (7-week-old, specific-pathogen-free; Orient Bio, Seongnam, Republic of Korea) were injected with 100 μl of the bacterial suspension along with CM14 (to achieve 1.4 mg/kg body weight) or 10% DMSO subcutaneously under the dorsal skin. Survival of the mice was monitored for 36 h as described previously^30^.

To examine the levels of blood biochemical parameters, pro-inflammatory cytokine production, and macrophage infiltration to the injection sites, the mice injected as described above were sacrificed at 7 h post infection to obtain blood and skin tissue samples, respectively. For blood biochemical analysis, the blood samples were collected using cardiac puncture in heparin-coated tube (IDEXX Laboratories, Westbrook, ME) and analyzed as described previously^30^. Breifly, the levels of TP, ALB, AST, ALT, BUN, and CREA in the blood plasma were measured by using a biochemistry autoanalyzer (Hitachi 7180 autoanalyzer, High-Technologies Corp., Tokyo, Japan). The remaining blood samples were fractionated by centrifugation for 10 min at 1,000 × *g* to obtain the blood plasma. Cytokine levels of IL-1β and IL-6 in the blood plasma were determined by ELISA using commercially available ELISA kits for IL-1β (R&D systems, Minneapolis, MN) and IL-6 (AbFrontier, Seoul, Republic of Korea). For immunohistochemical analysis, the mouse skin tissue samples around injection sites were embedded in optimum cutting temperature (O.C.T.) compound (Sakura Finetek, Torrance, CA) and stored at −80 °C. Frozen tissue samples were cryo-sectioned to a 20-μm thickness and then mounted on silane-coated slides (Muto Pure Chemicals, Tokyo, Japan). Tissue samples on slides were fixed with 80% acetone for 10 min, washed twice with PBS, and blocked in 5% normal goat serum (Sigma-Aldrich, St. Louis, MO) for 20 min. Slides were incubated with F4/80 antibody (1:100 dilution; Santa Cruz, Paso Robles, CA) for 2 h at room temperature. After washing three times with PBS, the slides were incubated with Alexa Fluor 488^®^-conjugated goat anti-rabbit secondary antibody (1:200 dilution; Thermo Fisher Scientific) for 1 h. Subsequently, all slides were incubated with DAPI solution (5 μg/ml; Thermo Fisher Scientific) in PBS for 5 min at room temperature. All immunofluorescence images were obtained by Eclipse Ts2^®^ fluorescence microscopy (Nikon, Tokyo, Japan), and colocalization of F4/80 with DAPI was analyzed by MetaMorph software (Universal Imaging, West Chester, PA).

### Protein purification, site-directed mutagenesis, and EMSA

The purification of recombinant HlyU was performed by affinity chromatography followed by size exclusion chromatography. Site-directed mutagenesis was performed using QuikChange Site-Directed Mutagenesis Kit as described previously^64^. For EMSA, the 264-bp [γ-^32^P]ATP-labeled DNA fragment of P_*rtxA*_ was amplified and incubated with the purified HlyU. Electrophoretic analysis of the DNA-protein complexes was performed as described previously^30^. When necessary, various concentrations of CM14 or DMSO were added to reaction mixture before incubation. As a control, a chemical randomly chosen from libraries that had no HlyU-inhibiting activity was added to the reaction mixture instead of CM14.

### Mass spectrometric analysis of the HlyU modification

The gel slices corresponding to HlyU protein treated with CM14 were destained and followed by in-gel reduction and alkylation of cysteine residues. The resulting samples were washed, digested by sequencing-grade trypsin, subjected to C18-SPE clean up, and reconstituted for LC-MS/MS analysis. The acquired datasets from LC-MS/MS experiment were initially searched to find the unknown cysteine modification and subjected to MS-GF + analysis^65^ to confirm the cysteine modification.

### Crystallization, structure determination, and refinement

HlyU protein was incubated with CM14 for 0.5 h at 4 °C and crystallized in a precipitation solution containing 0.1 M HEPES (pH 8.0), 20% (w/v) polyethylene glycol (PEG) 4 K and 10% (v/v) 2-propanol by hanging-drop vapor diffusion method at 14 °C. The HlyU-CM14 crystals were flash-frozen using 20% (w/v) sorbitol as a cryoprotectant in a nitrogen stream at −173 °C. An X-ray diffraction dataset was collected at Pohang Accelerator Laboratory beamline 5 C. The structure was determined and refined at a 2.1 Å resolution with an R factor of 23.8% and an R_free_ of 26.8%. Further details on the structure determination and refinement are given in Supplementary Table S4.

### Statistical analysis

Statistical analyses were performed as indicated in figure legends using GraphPad Prism 7.0 (GraphPad Software). For mouse lethality, mouse infection experiments were repeated twice to ensure reproducibility.

## Supplementary information

Supplementary Information
