# Supplementary material for: Small-molecule inhibitor of HlyU attenuates virulence of Vibrio species
Source: Sci Rep. 2019 Mar 13;9:4346. doi: 10.1038/s41598-019-39554-y (PMC6416295; doi:10.1038/s41598-019-39554-y)

**Small-molecule inhibitor of HlyU attenuates virulence of *Vibrio* species**

Zee-Won Lee^1,†^, Byoung Sik Kim^2,3,†^, Kyung Ku Jang^1,§^, Ye-Ji Bang^1,§§^, Suhyeon Kim^4^, Nam-Chul Ha^4^, Young Hyun Jung^5^, Hyun Jik Lee^5^, Ho Jae Han^5^, Jong-Seo Kim^6,7^, Jeesoo Kim^6,7^, Pramod K. Sahu^8,9^, Lak Shin Jeong^8^, Myung Hee Kim^3^, and Sang Ho Choi^1*^

^1^ National Research Laboratory of Molecular Microbiology and Toxicology, Department of Agricultural Biotechnology and Center for Food Safety and Toxicology, Seoul National University, Seoul, 08826, South Korea

^2^ Department of Food Science and Engineering, Ewha Womans University, Seoul, 03760, South Korea

^3^ Infection and Immunity Research Laboratory, Metabolic Regulation Research Center, Korea Research Institute of Bioscience and Biotechnology, Daejeon, 34141, South Korea

^4^ Department of Agricultural Biotechnology and Center for Food Safety and Toxicology, Seoul National University, Seoul, 08826, South Korea

^5^ Department of Veterinary Physiology, College of Veterinary Medicine, Research Institute for Veterinary Science, and BK21 PLUS Program for Creative Veterinary Science Research, Seoul National University, Seoul, 08826, South Korea

^6^ Center for RNA Research, Institute for Basic Science, Seoul National University, Seoul, 08826, South Korea

^7^ School of Biological Sciences, Seoul National University, Seoul, 08826, South Korea

^8^ Department of Pharmacy, Seoul National University, Seoul 08826, South Korea

^9^ Future Medicine Co., Ltd, Seoul, 06665, South Korea

^†^ These authors contributed equally to this work.

^§^ Present address: Department of Microbiology, New York University School of Medicine, New York, NY 10016, USA

^§§^ Present address: Department of Immunology, The University of Texas Southwestern Medical Center, 6000 Harry Hines Blvd., Dallas, TX 75390, USA.

^*^ Correspondence and requests for materials should be addressed to S. H. C. (email: choish@snu.ac.kr)

**Methods**

**Transcriptome analyses.** Total RNAs from the wild type and the *hlyU* mutant *V. vulnificus* MO6-24/O strains grown to *A*_600_ of 0.5 were isolated using RNAprotect^®^ Bacteria Reagent and miRNeasy^®^ Mini Kit (Qiagen, Valencia, CA) according to the manufacturer's procedure. The RNAs were further purified by removing genomic DNA using TURBO^TM^ DNase (Ambion, Austin, TX) and then cleaned up using RNeasy^®^ MinElute^TM^ Cleanup Kit (Qiagen). The quality of total RNAs was verified using Agilent 2100 Bioanalyzer and Agilent RNA 6000 Nano reagents (Agilent Technologies, Santa Clara, CA) by Chunlab (Seoul, Republic of Korea). The procedures for a strand-specific cDNA library construction and RNA-sequencing were conducted by Chunlab (Seoul). Briefly, mRNA was selectively enriched by depleting ribosomal RNAs using Ribo-Zero^TM^ rRNA Removal Kit (Epicentre, Madison, WI). Enriched mRNA was subjected to the cDNA library construction using TruSeq^®^ Stranded mRNA Sample Preparation Kit (Illumina, San Diego, CA) following manufacturer's instruction. The quality of cDNA libraries was evaluated as described above for the quality verification of total RNA, except that Agilent DNA 1000 Reagents (Agilent Technologies) was used instead. To ensure biological replication, RNAs were isolated from two independent cultures of the *V. vulnificus* strains, and two libraries were constructed per sample. Strand-specific paired-ended 100-nucleotide sequences from each cDNA library were obtained using HiSeq 2500 (Illumina). The raw sequencing reads were analyzed using CLC Genomics Workbench 5.5.1 (CLC Bio, Aarhus, Denmark) and mapped to the *V. vulnificus* MO6-24/O reference genome (GenBank^TM^ accession numbers: CP002469 and CP002470, www.ncbi.nlm.nih.gov). The expression level of each gene was defined using a RPKM (reads per kilobase of transcript per million mapped sequence reads) value, as described previously^1^. Quantile-normalized RPKM values were then statistically analyzed by *t*-tests to identify the differentially expressed genes (greater than 2-fold change with a *p*-value ≤ 0.05) from the *hlyU* mutant relative to the wild type.

Similarly, total RNAs from the wild type and the *hlyU* mutant *V. vulnificus* MO6-24/O strains grown to *A*_600_ of 0.5 in the presence of 20 μM of CM14 or 2% DMSO were isolated and subjected to the transcriptome analysis as described above. Heat maps were generated by the Gitools 2.3.1^2^ using the RPKM-fold change for each gene in the test samples. CLC Genomics workbench 11.0.1 software (CLC Bio) was used for a principal-component analysis of the whole-gene expression profiles of the samples.

**Construction of an *E. coli* reporter strain and high-throughput screening (HTS).** The *hlyU* ORF, amplified by PCR using a pair of primers HLYUS-F and HLYUS-R (Table S3), was subcloned into pBAD24^3^ carrying an arabinose-inducible promoter to yield pKK1306 (Table S1). The promoter region of VVMO6_00539, P*_VVMO6_00539_*, was amplified by PCR using a pair of primers 00539S-F and 00539S-R (Table S3) and then fused to the promoterless *lux* operon of pBBR_lux^4^ to create pZW1608, a HlyU-repressed reporter plasmid (Table S1). A reporter strain was constructed by transforming *E. coli* DH5α with pKK1306 and pZW1608. The *E. coli* reporter strain was grown to *A*_600_ of 0.5 in LB containing 0.0002% (w/v) L-(+) arabinose, 20 μg/ml chloramphenicol, and 100 μg/ml ampicillin. An aliquot (98 μl) of the culture was transferred to each well of a 96-well microtiter plate (Nunc™, Roskilde, Denmark) containing 2 μl of the small molecules to achieve 20 μM of each molecule or 2% DMSO (control) and incubated at 37°C with shaking. Luminescence and growth (*A*_600_) of the reporter strain in each well were measured after 3 h incubation using a microplate reader (Infinite™ M200 microplate reader, Tecan, Männedorf, Switzerland), and relative luminescence units (RLUs) were calculated by dividing luminescence with *A*_600_. The library information and screening results are summarized in Supplementary Table S2. Hit molecules inhibiting more than 10% of the HlyU activity were selected for further verification.

**Verification, structural confirmation, and determination of EC_50_ of CM14.** The promoter region of *rtxA*, P*_rtxA_*, was amplified by PCR using a pair of primers PrtxA-F and PrtxA-R (Table S3) and then fused to the promoterless *lux* operon of pBBR_lux^4^ to create pZW1609, a HlyU-activated reporter plasmid (Table S1). The *V. vulnificus* reporter strains were constructed by conjugally transferring either pZW1608 or pZW1609 into the wild-type *V. vulnificus* MO6-24/O and the *hlyU* mutant ZW141, respectively. The reporter strains were grown to *A*_600_ of 0.2 in LBS containing 3 μg/ml chloramphenicol and transferred to each well of a 96-well microtiter plate to achieve 20 μM of each hit molecule or 2% DMSO (control) as describe above. RLUs of the reporter strains were determined as described above but after 1.5 h incubation and then used to verify the HlyU-inhibiting activity of the hit molecules.

A molecule exhibiting the strongest HlyU-inhibiting activity was purchased from Vitas-M Laboratory (Moscow, Russian Federation) and named as CM14. The structure of CM14 was confirmed by ^1^H NMR and ^13^C NMR, using a Bruker AMX 500 spectrometer (Bruker, Karlsruhe, Germany), and by mass spectrometric analysis using Agilent 6530 Accurate-Mass Quadrupole Time-of-Flight (Q-TOF) mass spectrometer (Agilent Technologies) with a Jet Stream electrospray ionization source (ESI). The characteristics of CM14 were as follows: ^1^H NMR (500 MHz, CDCl_3_) δ 10.98 (s, 1 H), 7.78 (d with str, 1 H, *J* = 7.7 Hz), 7.65 (d with str, 2H, J = 7.2 Hz), 7.49-7.46 (m, 1H), 7.42-7.37 (m, 3 H), 7.31 (d with str, 1 H, 7.7 Hz), 7.27-7.25 (m, 1H), 7.19 (s, 1 H); ^13^C NMR (125 MHz, CDCl_3_) δ 159.93, 150.51, 149.67, 148.91, 133.01, 130.95, 130.89, 129.56, 128.67, 124.92, 123.45, 119.22, 117.63, 116.96, 107.72, 106.98, 89.96, 81.72; HRMS (ESI) Calcd for C_20_H_11_NO_3_S (M+H)^+^ 346.0538, found 346.0545.

To determine EC_50_ (the concentration of CM14 inhibiting the HlyU activity by 50%), the wild-type *V. vulnificus* reporter strain containing pZW1609 was grown to *A*_600_ of 0.2 in LBS containing 3 μg/ml chloramphenicol. An aliquot (98 μl) of the culture was transferred to each well of a 96-well microtiter plate (Nunc) containing 2 μl of various concentrations (10^-10^ to 10^-3^ M) of CM14 and incubated at 30°C with shaking. RLUs of the reporter strain in each well were measured after 1.5 h incubation using a microplate reader. The HlyU activities were expressed using the RLU observed in the absence of CM14 (in the presence of 2% DMSO) as 100%. The EC_50_ was calculated by plotting the relative HlyU activities versus the CM14 concentrations using GraphPad Prism 7.0 (GraphPad Software, San Diego, CA).

**Western blot and transcript analyses.** To evaluate the activity of CM14 *in vitro*, CM14 or 2% DMSO (control) was added to the *V. vulnificus* strains at *A*_600_ of 0.2. The strains were further grown to *A*_600_ of 0.5 and then used to analyze either the HlyU protein or the transcript levels of virulence genes. Cellular proteins of the *V. vulnificus* strains grown along with various concentrations of CM14 were isolated using cOmplete^TM^ Lysis-B EDTA-free buffer (Roche, Mannheim, Germany), and the total proteins in the clear cell lysates were quantified using a Bradford method. HlyU and DnaK in the cell lysates equivalent to 100 μg and 10 μg of total proteins, respectively, were detected using rabbit anti-*V. vulnificus* HlyU antibody and mouse anti-*E. coli* DnaK antibody (Enzo lifescience, Farmingdale, NY) by Western blot analysis as described elsewhere^5,6^.

Total RNAs from the *V. vulnificus* strains grown along with 20 μM of CM14 were isolated using RNeasy^®^ Mini Kit (Qiagen) and quantified using a Nano-Vue Plus spectrophotometer (GE Healthcare, Menlo Park, CA). To evaluate expression of specific genes, cDNA was synthesized from 1 μg of the total RNA by using iScript™ cDNA Synthesis Kit (Bio-Rad, Hercules, CA) and amplified by using the Chromo 4 real-time PCR detection system (Bio-Rad) with a pair of specific primers (Table S3). Relative expression levels of each gene were calculated by using the 16S rRNA expression level as the internal reference for normalization^7-10^.

**Protein purification, site-directed mutagenesis, and EMSA.** The ORF of *hlyU* amplified by PCR using a pair of primers HLYUP-F and HLYUP-R (Table S3) was digested with BamHI and XhoI and subcloned into pProEX-HTa (Invitrogen, Carlsbad, CA) to result in pZW1610 (Table S1). Plasmids pZW1611 and pZW1612 encoding mutant HlyUs in which Cys30 or Cys96 was replaced with serine, respectively, were created from pZW1610 using QuikChange Site-Directed Mutagenesis Kit (Agilent Technologies). The complementary mutagenic primers were used as listed in Table S3, and the mutations were confirmed by DNA sequencing. The His_6_-tagged HlyU proteins were then expressed in BL21 (DE3) and purified by affinity chromatography (Qiagen). The His_6_ tag was removed by treatment of recombinant tobacco etch virus (TEV) protease at room temperature, and the HlyU proteins were further purified by size exclusion chromatography using HiLoad Superdex 200 26/600 gel filtration column (GE Healthcare) equilibrated with buffer containing 300 mM NaCl, 20 mM Tris-HCl (pH 7.5), and 2 mM β-mercaptoethanol.

To express mutant HlyUs in which Cys30 or Cys96 was respectively replaced with serine, pZW1511 and pZW1512 were created in conjunction with the plasmid pZW1510^5^ as described above (Table S1). *E. coli* S17-1 λ *pir*^11^ harboring pZW1510, pZW1511, or pZW1512 was used as a conjugal donor to the *hlyU* mutant as described previously^7^.

For EMSA, the 264-bp DNA fragment of P*_rtxA_* was amplified by PCR using unlabeled PrtxA-EMSA-F and [γ-^32^P]ATP-labeled PrtxA-EMSA-R as primers (Table S3). The labeled P*_rtxA_* DNA (5 nM) was incubated with the purified HlyU for 0.5 h at 25°C in a 20-μl reaction mixture containing 1× binding buffer (10 mM Tris-Cl (pH 7.5), 50 mM KCl, 5 mM MgCl**_2_**, and 5% glycerol) and 0.1 μg of poly(dI-dC) (Sigma-Aldrich, St. Louis, MO). Electrophoretic analysis of the DNA-protein complexes was performed as described previously^5^. When necessary, either various concentrations of CM14 or DMSO was added to the reaction mixture before incubation. As a control, a chemical (20 μM) randomly chosen from libraries that had no HlyU-inhibiting activity was added to the reaction mixture instead of CM14.

**Mass spectrometric analysis of the HlyU modification.** For the *in vitro* reaction of HlyU and CM14, the purified HlyU protein was incubated with 10-fold excess amounts of CM14 for 0.5 h at 4°C. For the *in vivo* reaction, the HlyU protein was expressed in *E. coli* BL21 (DE3) cells in the presence of CM14 at 50 μM, and then purified as described above. The gel slices corresponding to HlyU protein treated with CM14 were destained in 50% acetonitrile (ACN) solution of 25 mM NH_4_HCO_3_ buffer for 10 min and then followed by in-gel reduction and alkylation of cysteine residues with dithiothreitol (DTT) and iodoacetamide (IAM). After washing out the excess reagents with 25 mM NH_4_HCO_3_ three times_,_ the resulting samples were digested by sequencing-grade trypsin at ratio of 1:50 (w/w) for overnight at 37°C. The digested peptides were subjected to C18-SPE clean up using 10 μl of ZipTip (Millipore, Temecula, CA) and then reconstituted with 25 mM NH_4_HCO_3_ for LC-MS/MS analysis. LC-MS/MS experiment with higher energy collisional dissociation (HCD) fragmentation mode was performed on Orbitrap Fusion Lumos mass spectrometry (Thermo Fisher Scientific, Waltham, MA) coupled with nanoACQUITY UPLC (Waters, Milford, MA) carrying an in-house packed capillary trap column (150 μm inside diameter, 3 cm long) and analytical column (75 μm inside diameter, 100 cm long) of 3 μm Jupiter C18 particles (Phenomenex Terrance, CA). The acquired datasets were initially searched by MODification via alignment (MODa)^12^, which is a blind search tool to find the unknown cysteine modification. Next, the datasets were subjected to MS-GF+ analysis at 10 ppm of precursor ion mass tolerance against *V. vulnificus* proteome database (GCF_000009745.1_ASM974v1) to confirm the cysteine modification.

**Crystallization, structure determination, and refinement.** For crystallization, HlyU was expressed as described previously^13^, with the exception of using *E. coli* C43 (DE3) instead. The HlyU protein (1.2 mM) was incubated with CM14 (10 mM) for 0.5 h at 4°C (molar ratio, 1:8.3), and then the mixture was centrifuged at 18,000 × *g* for 7 min to remove materials made during incubation. The clear fraction was crystallized in a precipitation solution containing 0.1 M HEPES (pH 8.0), 20% (w/v) polyethylene glycol (PEG) 4K and 10% (v/v) 2-propanol by hanging-drop vapor diffusion method at 14°C. The HlyU-CM14 crystals were flash-frozen using 20% (w/v) sorbitol as a cryoprotectant in a nitrogen stream at -173°C. An X-ray diffraction dataset was collected at Pohang Accelerator Laboratory beamline 5C and processed with the HKL2000 package^14^. The structure was determined using the MOLREP program in the CCP4 package by the molecular replacement method and a search model taken from the HlyU from *V. vulnificus* CMCP6 (PDB code: 3JTH)^13^. The final structure of the HlyU-CM14 was refined at a 2.1 Å resolution with an R factor of 23.8% and an R_free_ of 26.8% using the Coot and PHENIX refinement program^15^. Further details on the structure determination and refinement are given in Table S4.

**Data availability.** The RNA-sequencing data for the transcriptome analyses were deposited in NCBI Sequence Read Archive (SRA) database under accession numbers SRP128085 and PRJNA505764, respectively. The atomic coordinates and structure factors have been deposited in the Protein Data Bank (http://www.pdb.org) under PDB ID code 5ZNX.

**References**

1. Mortazavi, A., Williams, B. A., McCue, K., Schaeffer, L. & Wold, B. Mapping and quantifying mammalian transcriptomes by RNA-Seq. *Nat Methods* **5**, 621-628, doi:10.1038/nmeth.1226 (2008).
2. Perez-Llamas, C. & Lopez-Bigas, N. Gitools: analysis and visualisation of genomic data using interactive heat-maps. *Plos One* **6**, doi:10.1371/journal.pone.0019541 (2011).
3. Guzman, L. M., Belin, D., Carson, M. J. & Beckwith, J. Tight regulation, modulation, and high-level expression by vectors containing the arabinose P_BAD_ promoter. *J Bacteriol* **177**, 4121-4130 (1995).
4. Lenz, D. H. *et al.* The small RNA chaperone Hfq and multiple small RNAs control quorum sensing in *Vibrio harveyi* and *Vibrio cholerae*. *Cell* **118**, 69-82, doi:10.1016/j.cell.2004.06.009 (2004).
5. Jang, K. K. *et al.* Identification and characterization of *Vibrio vulnificus plpA* encoding a phospholipase A_2_ essential for pathogenesis. *J Biol Chem* **292**, 17129-17143, doi:10.1074/jbc.M117.791657 (2017).
6. Lim, J. G. & Choi, S. H. IscR is a global regulator essential for pathogenesis of *Vibrio vulnificus* and induced by host cells. *Infect Immun* **82**, 569-578, doi:10.1128/IAI.01141-13 (2014).
7. Bang, Y. J. *et al.* OxyR2 functions as a three-state redox switch to tightly regulate production of Prx2, a peroxiredoxin of *Vibrio vulnificus*. *J Biol Chem* **291**, 16038-16047, doi:10.1074/jbc.M115.710343 (2016).
8. Burke, A. K. *et al.* OpaR controls a network of downstream transcription factors in *Vibrio parahaemolyticus* BB22OP. *Plos One* **10**, 18, doi:10.1371/journal.pone.0121863 (2015).
9. Rui, H. P. *et al.* Role of alkaline serine protease, Asp, in *Vibrio alginolyticus* virulence and regulation of its expression by LuxO-LuxR regulatory system. *J Microbiol Biotechn* **19**, 431-438, doi:10.4014/jmb.0807.404 (2009).
10. Anthouard, R. & DiRita, V. J. Small-molecule inhibitors of *toxT* expression in *Vibrio cholerae*. *Mbio* **4**, doi:10.1128/mBio.00403-13 (2013).
11. Simon, R., Priefer, U. & Puhler, A. A broad host range mobilization system for *in vivo* genetic engineering: transposon mutagenesis in Gram-negative bacteria. *Nat Biotechnol* **1**, 784-791, doi:DOI 10.1038/nbt1183-784 (1983).
12. Na, S., Bandeira, N. & Paek, E. Fast multi-blind modification search through tandem mass spectrometry. *Mol Cell Proteomics* **11**, M111.010199, doi:10.1074/mcp.M111.010199 (2012).
13. Nishi, K. *et al.* Crystal structure of the transcriptional activator HlyU from *Vibrio vulnificus* CMCP6. *Febs Lett* **584**, 1097-1102, doi:10.1016/j.febslet.2010.02.052 (2010).
14. Otwinowski, Z. & Minor, W. Processing of X-ray diffraction data collected in oscillation mode. *Method Enzymol* **276**, 307-326, doi:Doi 10.1016/S0076-6879(97)76066-X (1997).
15. Adams, P. D. *et al.* PHENIX: a comprehensive Python-based system for macromolecular structure solution. *Acta Crystallogr D* **66**, 213-221, doi:10.1107/S0907444909052925 (2010).
16. Kim, S. *et al.* Complete genome sequence of *Vibrio parahaemolyticus* strain FORC_008, a foodborne pathogen from a flounder fish in South Korea. *Pathog Dis* **74**, doi:10.1093/femspd/ftw044 (2016).
17. Fullner, K. J. & Mekalanos, J. J. Genetic characterization of a new type IV-A pilus gene cluster found in both classical and El Tor biotypes of *Vibrio cholerae*. *Infection and Immunity* **67**, 1393-1404 (1999).
18. Dumon-Seignovert, L., Cariot, G. & Vuillard, L. The toxicity of recombinant proteins in *Escherichia coli*: a comparison of overexpression in BL21(DE3), C41(DE3), and C43(DE3). *Protein Expres Purif* **37**, 203-206, doi:10.1016/j.pep.2004.04.025 (2004).
19. Goo, S. Y. *et al.* Identification of OmpU of *Vibrio vulnificus* as a fibronectin-binding protein and its role in bacterial pathogenesis. *Infect Immun* **74**, 5586-5594, doi:10.1128/IAI.00171-06 (2006).

**SI Figure legends**

**Supplementary Figure S1. Genes regulated by HlyU.** Genes expressed differentially in the isogenic *hlyU* mutant (fold change ≥ 2; *p* ≤ 0.05) were identified by transcriptome analyses and considered as a HlyU regulon. (**a**) Among them, 7 genes whose expressions were confirmed by qRT-PCR are presented. Each column represents the mRNA expression level in the *hlyU* mutant relative to that in the wild type. Means and standard deviations (SD) were calculated from at least three independent experiments. Locus tags are based on the database of the *V. vulnificus* MO6-24/O genome (GenBank^TM^ accession numbers: CP002469 and CP002470), and the products of the 7 genes are presented on the right. (**b**,**c**) EMSAs for the binding of HlyU to the upstream regions of VVMO6_00539 (**b**) and VVMO6_03281 (**c**). Each radioactively-labeled probe DNA (5 nM) was incubated with increasing amounts of HlyU as indicated. For competition analysis, the same but unlabeled probe DNA was used as a self-competitor DNA. Various amounts of the self-competitor DNA were added to a reaction mixture containing the 5 nM labeled probe DNA before the addition of 300 nM (**b**) and 250 nM (**c**) HlyU as indicated. The DNA-HlyU complexes were separated as described in the legend to Fig. 5. B, bound DNA; F, free DNA. (**d**,**e**) Effects of CM14 on the expression of the HlyU-repressed genes. The *V. vulnificus* strains were grown in the presence of CM14 (20 μM) or 2% DMSO (control) to *A*_600_ of 0.5. The transcript levels of VVMO6_00539 (**d**) and VVMO6_03281 (**e**) in the total RNA of the cells were quantified by qRT-PCR and expressed using each transcript level of the wild type in the presence of DMSO as 1. Error bars represent the SD from biological triplicates. Statistical significance was determined by the Student's *t*-test (*, *p* < 0.05; *ns*, not significant). WT, wild type; *hlyU*, *hlyU* mutant.

**Supplementary Figure S2. Mass spectrum of HlyU and HlyU_C30S_ peptide in the presence of CM14 and close-up view around Cys30 in HlyU structure.** (**a**) MS/MS spectrum of Cys30-modified peptide (RLQIL**C#**MLHNQELSVGELCAK) from the freshly purified HlyU protein expressed in the *E. coli* cells treated with 50 μM of CM14. C# indicates the mass shift of C_9_H_6_O (# = + 130.042 Da) by the cysteine modification. Both N- and C-terminal fragment ion series are represented as b and y series, respectively (e.g. b5, b6, b7… and y1, y2, y3…), and the annotated fragment ions are marked in the inserted peptide sequence. The observed precursor ion (monoisotopic m/z 862.446) in the inserted high resolution MS spectrum matched exactly with a theoretical m/z (862.439). (**b**) MS/MS spectrum of RLQIL***S***MLHNQELSVGELCAK from the CM14-treated HlyU_C30S_ mutant protein. ***S*** indicates the Ser30 that replaced the Cys30. Both N- and C-terminal fragment ion series are represented as b and y series, respectively (e.g. b5, b6, b7… and y1, y2, y3…), and the annotated fragment ions are marked in the inserted peptide sequence. The observed precursor ion (monoisotopic m/z 813.775) in the inserted high resolution MS spectrum matched exactly with a theoretical m/z (813.766). (**c**) Close-up view around the Cys30 residue in the HlyU structure^13^ (PDB code: 3JTH). Sulfur, oxygen, and nitrogen atoms of the residues are shown as yellow, red, and blue, respectively.

**Supplementary Figure S3. Sequence alignment of HlyU from different *Vibrio* species.** The HlyU amino acid sequences retrieved from NCBI protein database (accession numbers: WP_011078600.1 for *V. vulnificus* HlyU, WP_005461077.1 for *V. parahaemolyticus* HlyU, WP_001888967.1 for *V. cholerae* HlyU, WP_005381940.1 for *V. alginolyticus* HlyU, WP_011261241.1 for *V. fischeri* HlyU, WP_005447923.1 for *V. harveyi* HlyU, WP_026028082.1 for *V. anguillarum* HlyU, WP_001053147.1 for *V. mimicus* HlyU, WP_006075375.1 for *V. shilonii* HlyU, WP_004397917.1 for *V. nigripulchritudo* HlyU, WP_001888967.1 for *V. albensis* HlyU, WP_004735028.1 for *V. splendidus* HlyU, and WP_004744856.1 for *V. tubiashii* HlyU) were aligned using the Clustal Omega program. Identical (asterisks), highly conserved (double dots), and conserved (dots) sequences are indicated. The positions of Cys30 and Cys96 of *V. vulnificus* HlyU are highlighted as red and blue, respectively.

**Supplementary Figure S4. Effect of CM14 on growth of other *Vibrio* species*.*** *V. parahaemolyticus* (**a**), *V. alginolyticus* (**b**), and *V. cholerae* (**c**) were grown at 37°C in LBS, tryptic soy broth supplemented with 1% (w/v) NaCl, and LB, respectively, along with various concentrations of CM14 or 2% DMSO (control). Their growth was monitored at 1 h intervals using a microplate reader.

**Supplementary Figure S5. Effects of CM14 on HlyU regulon expression.** (**a**) Genes differentially expressed in DMSO-treated *hlyU* mutant, CM14-treated *hlyU* mutant, and CM14-treated wild type relative to those expressed in DMSO-treated wild type (fold change ≥ 2; *p* ≤ 0.05) were identified by transcriptome analyses, and the fold changes of the expression of these genes are shown in the heat map with colors representing the log_2_ RPKM ratio. Locus tags are based on the database of the *V. vulnificus* MO6-24/O genome (GenBank^TM^ accession numbers: CP002469 and CP002470), and the products of the genes are presented on the right. (**b**) Principal-component analysis of the whole-gene expression profiles of the samples. Each symbol represents the transcriptome of a single sample from two biological replicates per sample group. WT, wild type; *hlyU*, *hlyU* mutant.

**Supplementary Figure S6. Effects of CM14 treatment after infection of *V. vulnificus* to host cells.** INT-407 cells were infected with the *V. vulnificus* strains at a multiplicity of infection of 10 and then treated with various concentrations of CM14 as indicated after 0.5 h (**a**) or 1 h (**b**) of infection. Cytotoxicity was determined using LDH activities released from the cells after 2.5 h incubation and expressed using the LDH activity from the cells completely lysed by 5% Triton X-100 as 100%. Error bars represent the SD from the representative of three independent experiments. Statistical significance was determined by one-way ANOVA (***, *p* < 0.0005; *ns*, not significant). WT, wild type; *hlyU*, *hlyU* mutant.

**Supplementary Figure S7. Original images for Figure 2c.** Clear lysate samples of biological duplicate were prepared and resolved on SDS-PAGE gels as described in Methods. After electrophoresis, the gels were cropped based on the molecular weight of HlyU (12 kDa) and DnaK (69 kDa). (**a**) Lower parts of the gels were transferred together to a nitrocellulose membrane to detect HlyU using rabbit polycolonal anti-*V. vulnificus* HlyU antibody (1:300; ^5^) and goat polyclonal anti-rabbit IgG antibody conjugated with horseradish peroxidase (HRP) (1:10,000; Sigma). (**b**) Upper parts of the gels were transferred to another nitrocellulose membrane to detect DnaK using mouse monoclonal anti-*E. coli* DnaK antibody (1:10,000; Enzo lifescience) and goat polyclonal anti-mouse IgG antibody conjugated with alkaline phosphatase (1:10,000; Sigma). Visualization was followed with ECL Select^TM^ Western blotting detection reagent (GE Healthcare) for HlyU, and 5-bromo-4-chloro-3-indolyl phosphate/nitroblue tetrazolium (Sigma) for DnaK. Upper and lower black dashed boxes in each panel show biological replicates, as indicated. Molecular size markers (Bio-Rad) are shown in kDa. The cropped images shown in Fig. 2c were indicated with red dashed boxes.

**Supplementary Figure S8. Original images for Figure 5 and Figure 6b.** The HlyU-DNA complexes were separated by electrophoresis on 6% nondenaturing polyacrylamide gels. The gels were visualized using a Typhoon FLA 7000 phosphorimager (GE healthcare). The cropped images shown in Fig. 5 and Fig. 6b were indicated as red dashed boxes in (**a**), (**b**), (**c**), (**d**), and (**e**), respectively. In (**e**), the unrelated sample lanes were marked by cross lines. B, bound DNA; F, free DNA.

**Table S1.** Bacterial strains and plasmids used in this study

| **Strain or plasmid** | **Relevant characteristics^a^** | **Reference or source** |
| --- | --- | --- |
| **Bacterial strains** |  |  |
| *V. vulnificus* |  |  |
| MO6-24/O | Wild type; clinical isolate; virulent | Laboratory collection |
| ZW141 | MO6-24/O with Δ*hlyU* | ^5^ |
|  |  |  |
| *V. parahaemolyticus* |  |  |
| FORC_008 | Wild type; clinical isolate; virulent | ^16^ |
|  |  |  |
| *V. alginolyticus* |  |  |
| ATCC17749 | Wild type; virulent | Korean Collection for Type Cultures |
|  |  |  |
| *V. cholerae* |  |  |
| El Tor N16961 | Wild type; clinical isolate; virulent | ^17^ |
|  |  |  |
| *E. coli* |  |  |
| DH5α | *supE44 ΔlacU169* (*Φ80 lacZ Δ*M15) *hsdR17 recA1 endA1 gyrA96 thi-1 relAI* | Laboratory collection |
| BL21 (DE3) | *F*^-^, *ompT*, *hsdS* (r_B_^-^, m_B_^-^), *gal dcm* (DE3) | Laboratory collection |
| C43 (DE3) | *F*^-^, *ompT*, *hsdS* (r_B_^-^, m_B_^-^), *gal dcm* (DE3) | ^18^ |
| S17-1 λ *pir* | Tc::Mu-Km::Tn7;Tp^r^ Sm^r^; host for π-requiring plasmids | ^11^ |
|  |  |  |
| **Plasmids** |  |  |
| pBBR_lux | Broad host range vector with promoterless *luxCDABE*; Cm^r^ | ^4^ |
| pZW1608 | pBBR_lux with P*_VVMO6_00539_*; Cm^r^ | This study |
| pZW1609 | pBBR_lux with P*_rtxA_*; Cm^r^ | This study |
| pBAD24 | Expression vector with the P*_BAD_* promoter; Ap^r^ | ^3^ |
| pKK1306 | pBAD24 with *hlyU*; Ap^r^ | This study |
| pProEX-HTa | His_6_-tag fusion protein expression vector; Ap^r^ | Invitrogen |
| pZW1610 | pProEX-HTa with *hlyU*; Ap^r^ | This study |
| pZW1611 | pProEX-HTa with mutant *hlyU* encoding HlyU-C30S; Ap^r^ | This study |
| pZW1612 | pProEX-HTa with mutant *hlyU* encoding HlyU-C96S; Ap^r^ | This study |
| pJH0311 | 0.3-kb MCS of pUC19 cloned into pCOS5; Ap^r^, Cm^r^ | ^19^ |
| pZW1510 | pJH0311 with *hlyU*; Ap^r^, Cm^r^ | ^5^ |
| pZW1511 | pJH0311 with mutant *hlyU* encoding HlyU-C30S; Ap^r^, Cm^r^ | This study |
| pZW1512 | pJH0311 with mutant *hlyU* encoding HlyU-C96S; Ap^r^, Cm^r^ | This study |

^a^ Tp^r^, trimethoprim resistant; Sm^r^, streptomycin resistant; Cm^r^, chloramphenicol-resistant; Ap^r^, ampicillin-resistant.

**Table S2.** Small molecule screening information

| Category | Parameter | Description |
| --- | --- | --- |
| Assay | Type of assay | Whole organism (*E. coli* cells) |
|  | Target | HlyU protein |
|  | Primary measurement | Detection of bioluminescence and absorbance at 600 nm (*A*_600_) |
|  | Key reagents | L-(+)-arabinose for the induction of *hlyU* gene |
|  | Assay protocol | Refer to the Methods section |
|  | Additional comments | *E. coli* cells contain the reporter plasmid with *lux* operon that is fused to a HlyU-repressed promoter |
| Library | Library size | Total of 8,385 molecules (8,364 used for initial screening, plus additional 21 structural homologues)  Arrayed in 96-well plates as single compounds at 20 μM in DMSO (total 80 compounds per plate, leaving first and second columns empty for control samples) |
|  | Library composition | Structure-representative library  (contains diverse molecules from different PharmaCore structures (~1,000); considered for drug-likeness and solubility; all compounds confirmed as >85% pure by LC-MS analysis) |
|  | Source | Korea Chemical Bank |
|  | Additional comments | For more information about the library, refer to http://www.chembank.org/ |
| Screen | Format | 96-well optical bottom plate w/Lid Black (Thermo Fisher Scientific) |
|  | Concentration(s) tested | 20 μM compound, 2% DMSO |
|  | Plate controls | Negative control: DMSO-treated *E. coli* cells plus L-(+)-arabinose; Positive control: DMSO-treated *E. coli* cells without L-(+)-arabinose |
|  | Reagent/compound dispensing system | Manual |
|  | Detection instrument and software | Infinite™ M200 microplate reader (Tecan, Männedorf, Switzerland); Tecan i-Control ver 1.4.9.0 |
|  | Assay validation/QC | For screening validation, every plate contained positive control samples that do not express HlyU protein  Z-factor: 0.79; Z'-factor: 0.83;  SD of positive control: 320.48; SD of negative control: 33.15 |
|  | Correction factors | Luminescence was normalized against *E. coli* cell growth (*A*_600_) to obtain relative luminescence unit (RLU) |
|  | Normalization | % HlyU-inhibition = 100×(y-z)/(y-x), where x is the average RLU of the positive control samples, y is the average RLU of the negative control samples, and z is the RLU of each molecule-treated sample |
|  | Additional comments | Values were measured after 3 h of incubation at 37°C |
| Post-HTS analysis | Hit criteria | % HlyU-inhibition >10 (%) |
|  | Hit rate | 0.036% (3 of 8,385 molecules) |
|  | Additional assay(s) | Verification of initial hits in the original (Fig. 1b) and secondary assays (Fig. 1c and d) |
|  | Confirmation of hit purity and structure | A hit molecule was repurchased from Vitas-M Laboratory, and the structure of CM14 was confirmed by ^1^H NMR, ^13^C NMR, and mass spectrometric analyses |
|  | Additional comments |  |

**Table S3.** Oligonucleotides used in this study

| **Oligonucleotide** | **Oligonucleotide sequence, 5’** **→3’^a, b^** | **Location^c^** | **Use** |
| --- | --- | --- | --- |
| HLYUS-F | GCTAGCTAGAGGACCAACACATG | Upstream of VVMO6_02507 | Amplification of *hlyU* ORF for reporter strain construction |
| HLYUS-R | GGTACCTTATTCTTCGCAATAAAG | VVMO6_02507 |  |
| 00539S-F | GAGCTCATTTGTTTAAGCGTGTAAAGC | Upstream of VVMO6_00539 | Amplification of VVMO6_00539 upstream region |
| 00539S-R | ACTAGTTTTTCGTAGCTGCTCAATTTGTAAT | VVMO6_00539 |  |
| 03281S-F | GGCCAAGTAATTTTATCGTTTTCATGATAC | Upstream of VVMO6_03281 | Amplification of VVMO6_03281 upstream region |
| 03281S-R | ACTAGTAAAGGGGTTGTGAGTCGATAATCA | VVMO6_03281 |  |
| PrtxA-F | GAGCTCGAATCAAATAAAATGGC | P*_rtxA_* | Amplification of P*_rtxA_* |
| PrtxA-R | ACTAGTTATTTTTTTGATCCTGGCCTAC | P*_rtxA_* |  |
| RTXA-qRT-F | TAGCGGCGACAATGAAACCT | VVMO6_03947 | qRT-PCR |
| RTXA-qRT-R | CCCATCACCGCAAGGGTATT | VVMO6_03947 |  |
| VVHA-qRT-F | ACAGCTGGTTCCAGAGTTGG | VVMO6_03881 |  |
| VVHA-qRT-R | AACGGGTTTCACCCAAAGGT | VVMO6_03881 |  |
| PLPA-qRT-F | TTGTTGGTATCGAACGGGCA | VVMO6_03257 |  |
| PLPA-qRT-R | CGAGCTCCACCAATAACCGT | VVMO6_03257 |  |
| 00539-qRT-F | CAGGCGGGTTATTACGGTGT | VVMO6_00539 |  |
| 00539-qRT-R | TTTCTTGCTCGCTCTTTGCG | VVMO6_00539 |  |
| 01210-qRT-F | AAAACCACCTTGCTCAACGC | VVMO6_01210 |  |
| 01210-qRT-R | ATCCAATGTGGCTGGGTCAG | VVMO6_01210 |  |
| 03281-qRT-F | GGCATCAGTTTGGCGAAGTC | VVMO6_03281 |  |
| 03281-qRT-R | TATACGCTACGTTGGACAGGC | VVMO6_03281 |  |
| 03824-qRT-F | ATCTTCGATTTGGCCGTTGC | VVMO6_03824 |  |
| 03824-qRT-R | CTCGCGGTATGGACGTAGAC | VVMO6_03824 |  |
| VPEXSA-qRT-F | TTTCCGGCATGAAAACGCTG | FORC8_1324 |  |
| VPEXSA-qRT-R | GGTTCGGTTAGTTTGCTGCG | FORC8_1324 |  |
| VP1668-qRT-F | ACCAATTTCGCTTGGCGTTC | FORC8_1354 |  |
| VP1668-qRT-R | AACGGTTACGTCTACGTCCG | FORC8_1354 |  |
| VPVOPQ-qRT-F | CGCCGATAGCAAAAGAAGCC | FORC8_1342 |  |
| VPVOPQ-qRT-R | TGGCCCTTGTTTGAGTTGGT | FORC8_1342 |  |
| VPVOPS-qRT-F | TAGAACGCGATTACCGTGGG | FORC8_1338 |  |
| VPVOPS-qRT-R | TTACCGAGGTCTTTGTCCGC | FORC8_1338 |  |
| VPVOPR-qRT-F | CAAAACGTGCTCAATGGCGA | FORC8_1340 |  |
| VPVOPR-qRT-R | TTTGGAAGACGAGGTGTCGG | FORC8_1340 |  |
| VALEXSA-qRT-F | TGGTGCCAGCTTAACCACTT | N646_0765 |  |
| VALEXSA-qRT-R | ACGATGCTCATCTTGCTCGT | N646_0765 |  |
| VAL1668-qRT-F | TTAGCGGAAGTGGTTGGCTT | N646_0736 |  |
| VAL1668-qRT-R | GTACCTAACAGGTGGTCGCC | N646_0736 |  |
| VALVOPQ-qRT-F | CACCGATAGCAAAAGAGGCA | N646_0746 |  |
| VALVOPQ-qRT-R | CTCACCTTGCATGAGTTGTT | N646_0746 |  |
| VALVOPS-qRT-F | TACCAAATCAACGCAGCGAC | N646_0751 |  |
| VALVOPS-qRT-R | ACCGATTGTGGGCTACCTTC | N646_0751 |  |
| VALVOPR-qRT-F | ACAAACGGCAGCCTCTTCTT | N646_0748 |  |
| VALVOPR-qRT-R | TGACCCCTTAACACCGAACG | N646_0748 |  |
| HLYA-qRT-F | CGTCAACAATACGCGACAGC | VC_A0219 |  |
| HLYA-qRT-R | CTCAGCGGGCTAATACGGTT | VC_A0219 |  |
| TLH-qRT-F | GTCAACCACGATTTTGCCGA | VC_A0218 |  |
| TLH-qRT-R | GATGCGGTTGGACTGAACCT | VC_A0218 |  |
| RTXAVC-qRT-F | AAAGCCAACGTCGTGACTCATG | VC_1451 |  |
| RTXAVC-qRT-R | ATCGGAAATCAACAACCCTACCGT | VC_1451 |  |
| PrtxA-EMSA-F | TCAAATAAAATGGCGGGTGT | P*_rtxA_* | Amplification of P*_rtxA_* |
| PrtxA-EMSA-R | CCTTCAAAAACGCTGCAAT | P*_rtxA_* |  |
| HLYUP-F | GGTGGATCCAATGAACTTAAAAGATATGG | VVMO6_02507 | Amplification of *hlyU* ORF for protein purification |
| HLYUP-R | GGTCTCGAGTTATTCTTCGCAATAAAG | VVMO6_02507 |  |
| HLYUC30S-F | GACGCCTGCAAATCTTA**TCC**ATGCTACACAATCAAGAG | VVMO6_02507 | Construction of HlyU-C30S mutant |
| HLYUC30S-R | CTCTTGATTGTGTAGCAT**GGA**TAAGATTTGCAGGCGTC | VVMO6_02507 |  |
| HLYUC96S-F | GCACAGTCTTTAT**TCC**GAAGAATAATGCTTTTGCGTGCC | VVMO6_02507 | Construction of HlyU-C96S mutant |
| HLYUC96S-R | GGCACGCAAAAGCATTATTCTTC**GGA**ATAAAGACTGTGC | VVMO6_02507 |  |

^a^ Mutated nucleotide is shown in bold.

^b^ Regions of oligonucleotides not complementary to the corresponding genes are underlined.

^c^ Nucleotide hybridization site.

**Table S4.** Statistics for X-ray data collection and refinement

|  | HlyU-CM14 |
| --- | --- |
| **Data collection** |  |
| Beamline | PAL 5C |
| Wavelength (Å) | 0.97960 |
| Space group | *P*4_1_2_1_2 |
| Cell dimensions |  |
| *a, b, c* (Å) | 35.1, 35.1, 180.4 |
| α, β, γ (˚) | 90, 90, 90 |
| Resolution (Å) | 50-2.1 (2.14-2.10)^a^ |
| R_merge_^b^ | 0.208 (0.114) |
| *I/σI* | 24.12 (5.64) |
| Completeness (%) | 97.2 (92.2) |
| Redundancy | 19.7 (9.5) |
| **Refinement** |  |
| Resolution (Å) | 32.74-2.1 |
| No. of reflections | 6758 |
| R_work_/R_free_^c^ | 0.238/0.268 |
| No. of total atoms | 789 |
| Wilson B-factor (Å) | 22.60 |
| RMSD |  |
| Bond lengths (Å) | 0.004 |
| Bond angles (˚) | 0.81 |
| Ramachandran plot |  |
| Favored (%) | 94.6 |
| Allowed (%) | 5.4 |
| Outliers (%) | 0 |

^a^ Values in parentheses are for the highest-resolution shell.

^b^ *R*_merge_ = Σ_hkl_Σ_i_|*I_i_*(*hkl*) – [*I*(*hkl*)]|/Σ_hkl_Σ_i_I*_i_*(*hkl*)*,* where I_i_(*hkl*) is the intensity of the *i*th observation of reflection *hkl* and [I(*hkl*)] is the average intensity of the *i* observations.

^c^ *R*_free_ calculated for a random set of 10% of reflections not used in the refinement.

**Supplementary Figure S1**


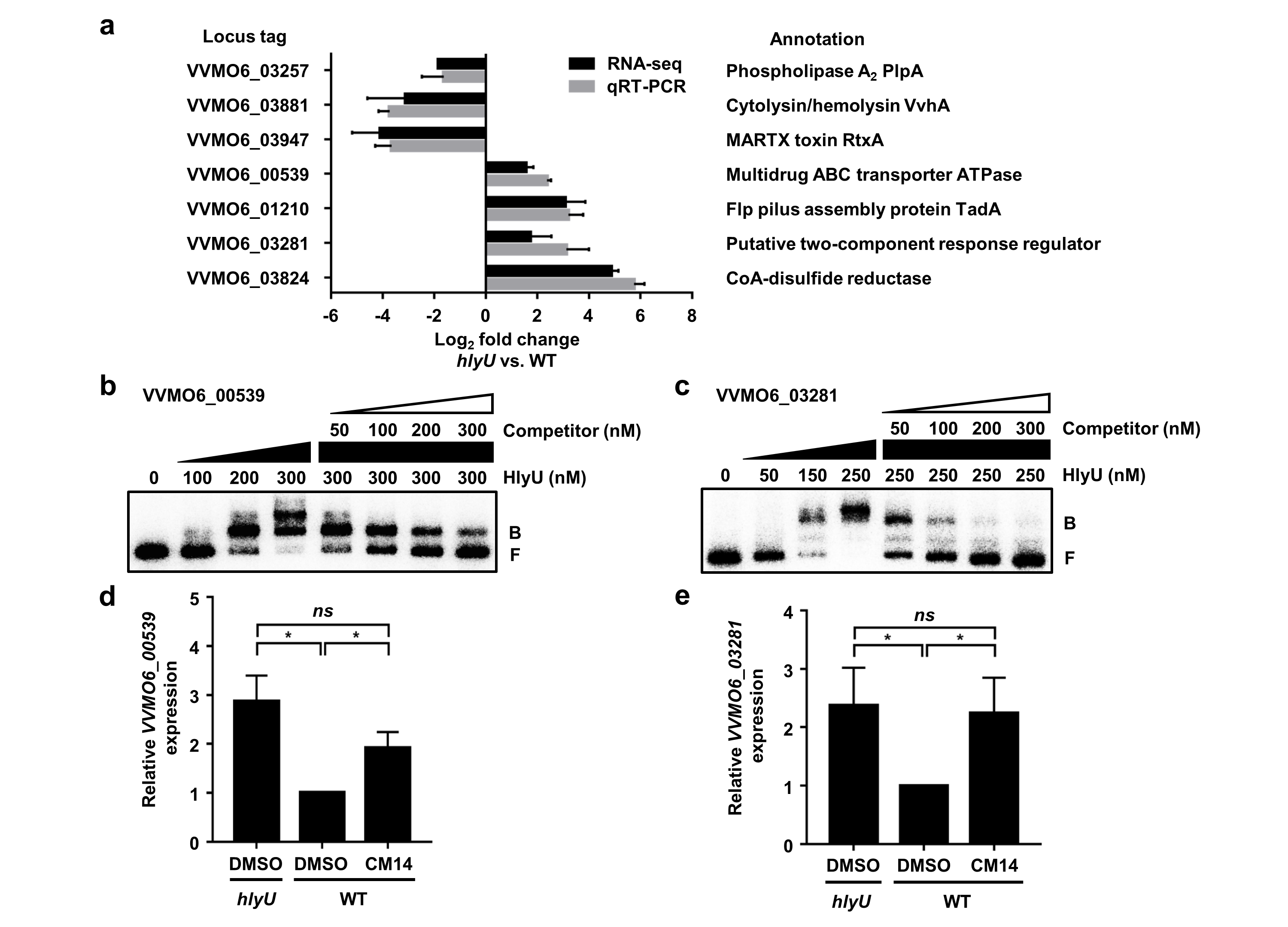


**Supplementary Figure S2**


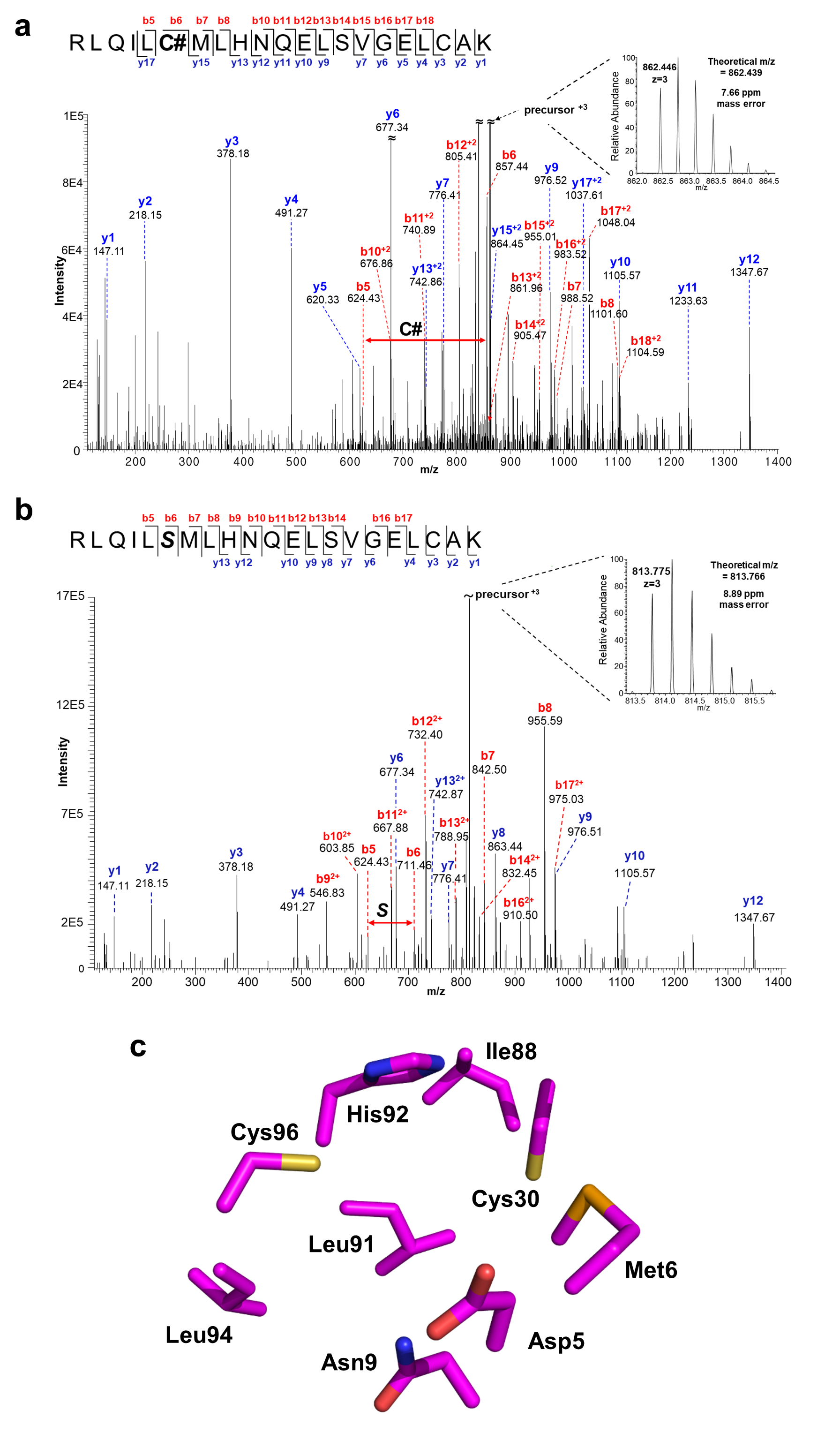


**Supplementary Figure S3**


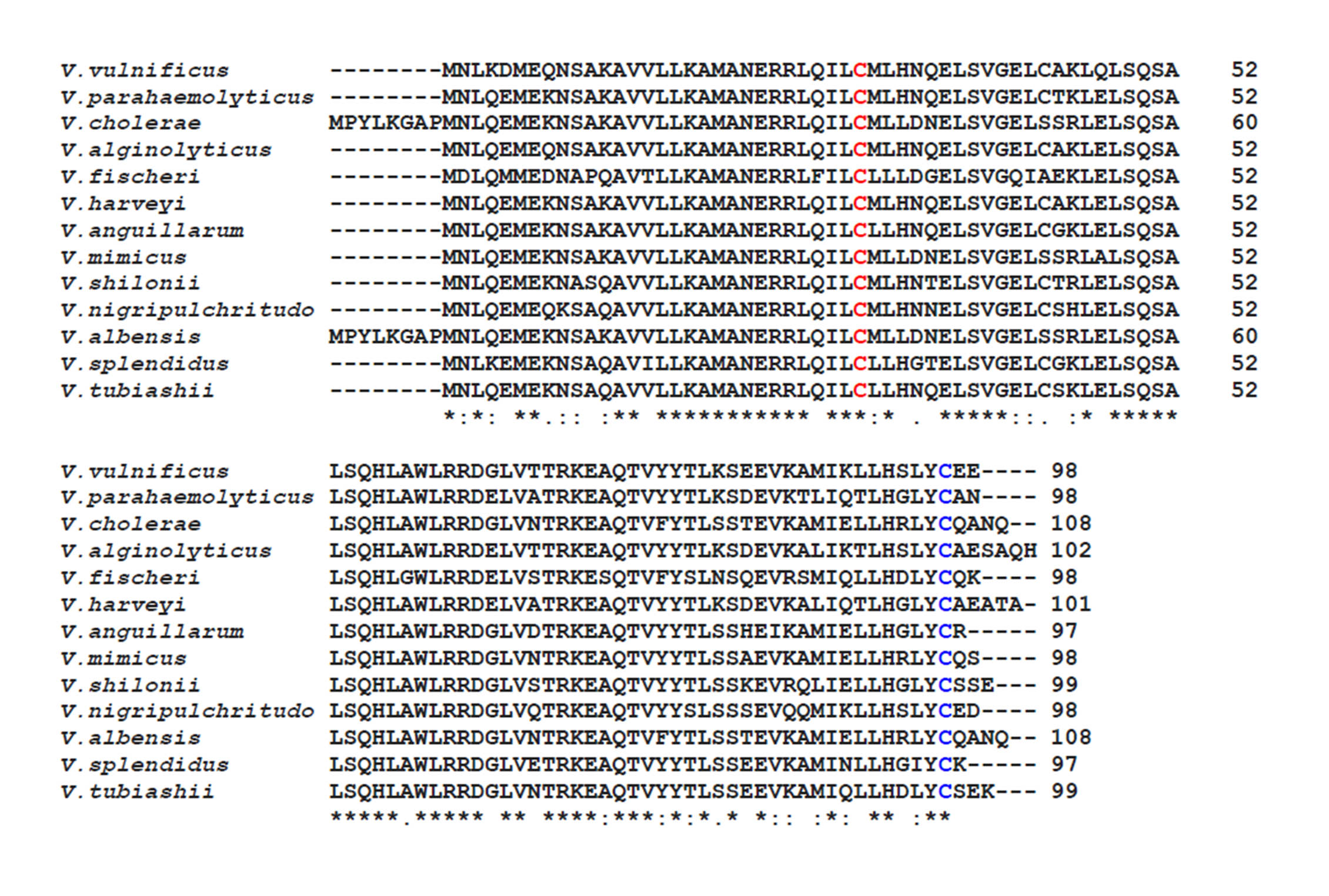


**Supplementary Figure S4**


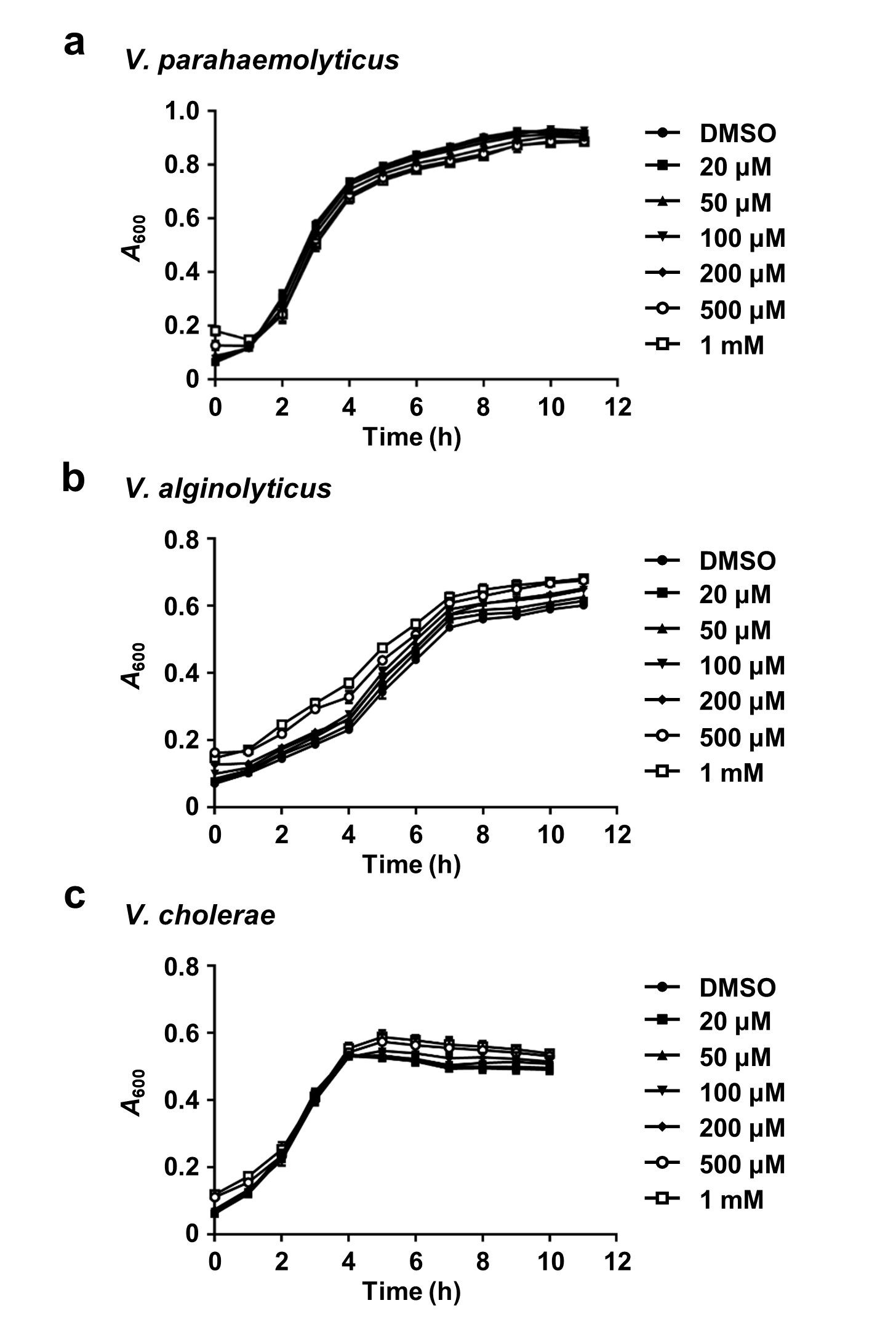


**Supplementary Figure S5**


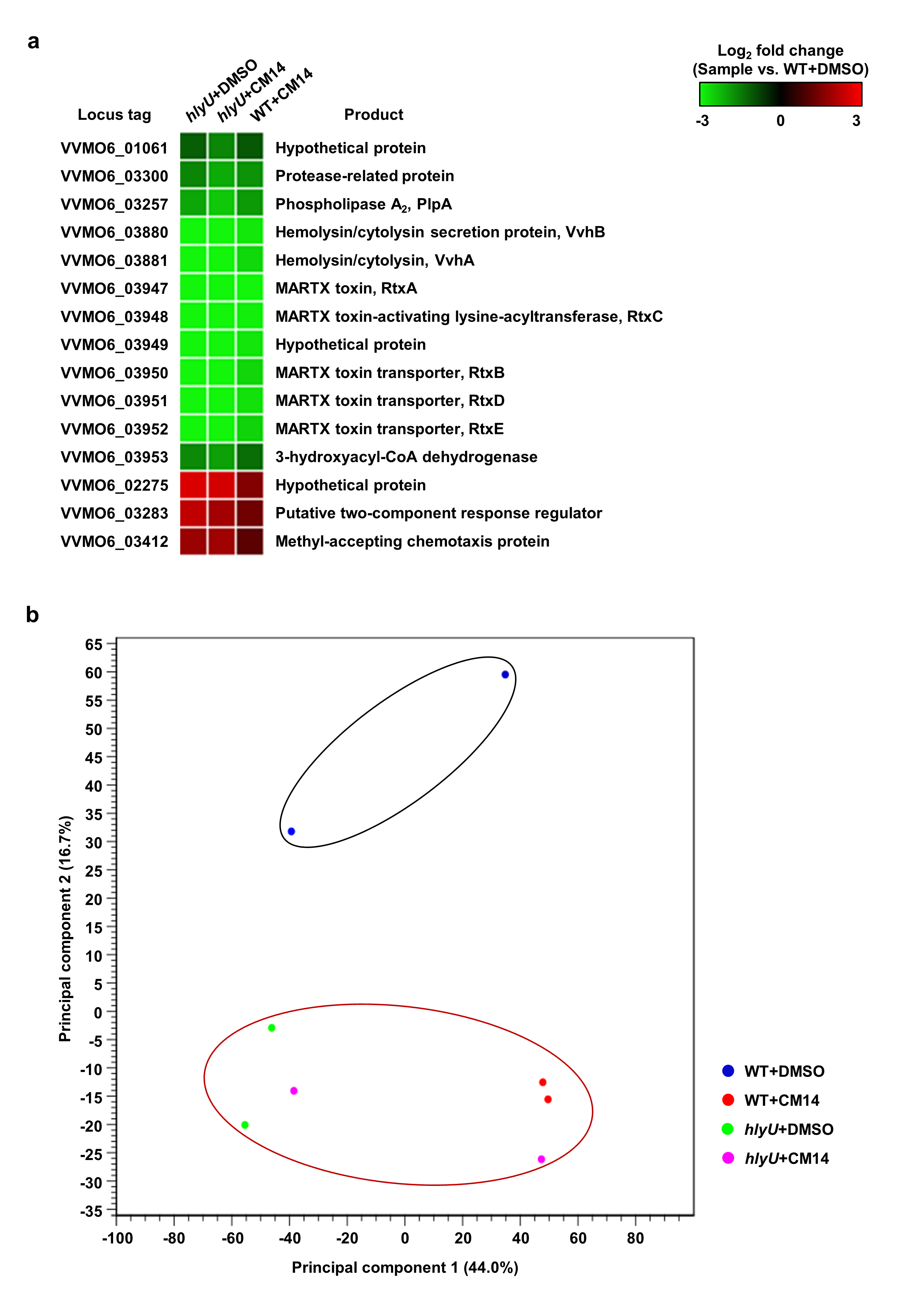


**Supplementary Figure S6**


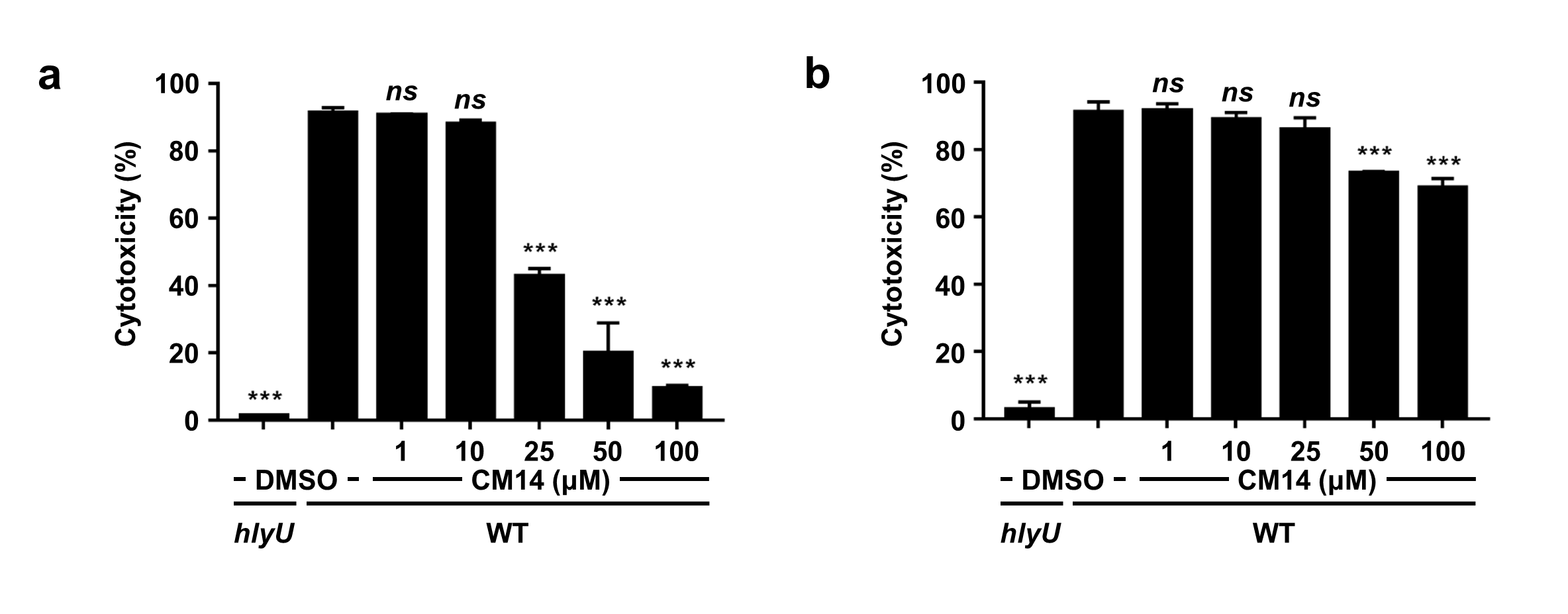


**Supplementary Figure S7**


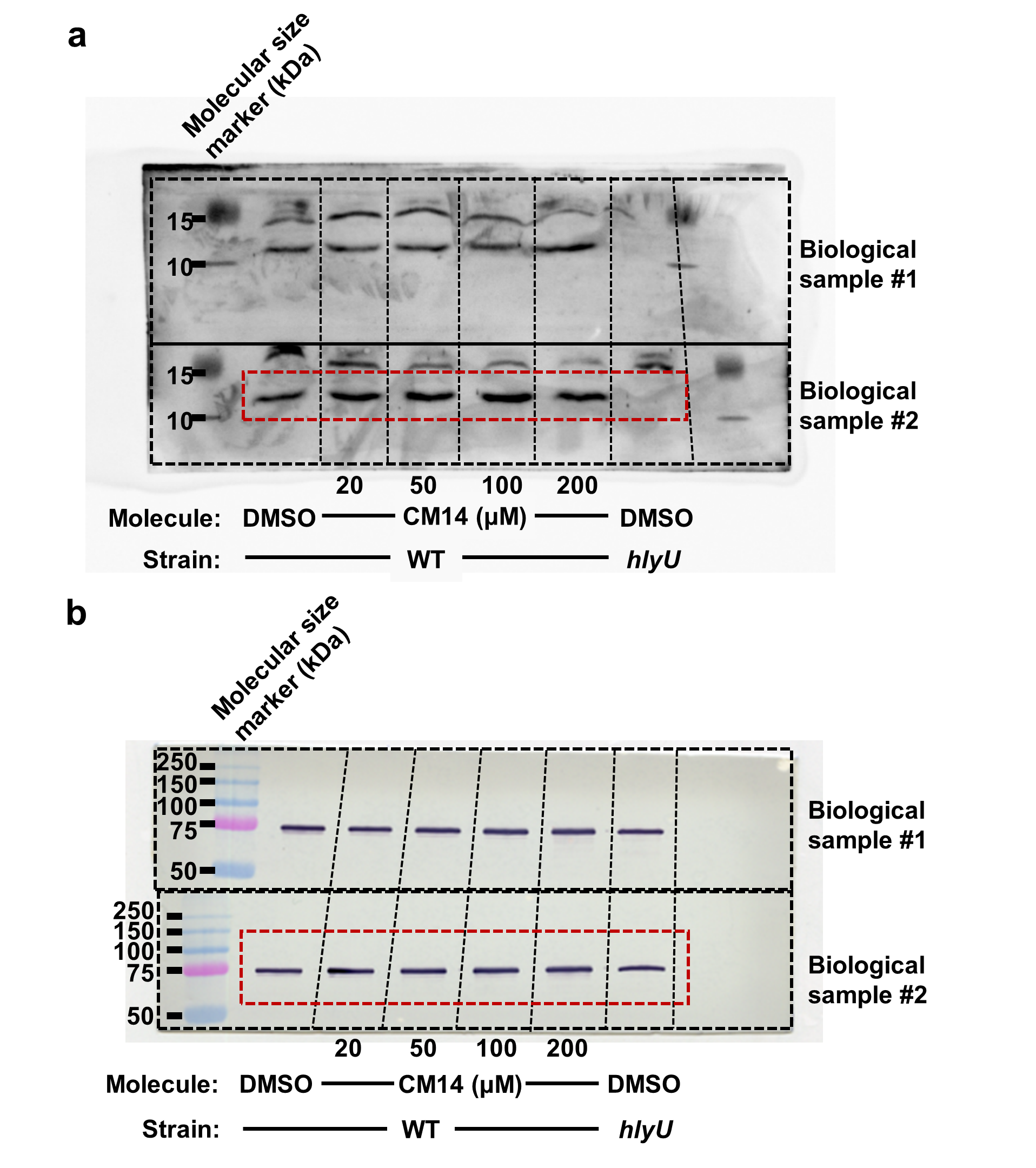


**Supplementary Figure S8**


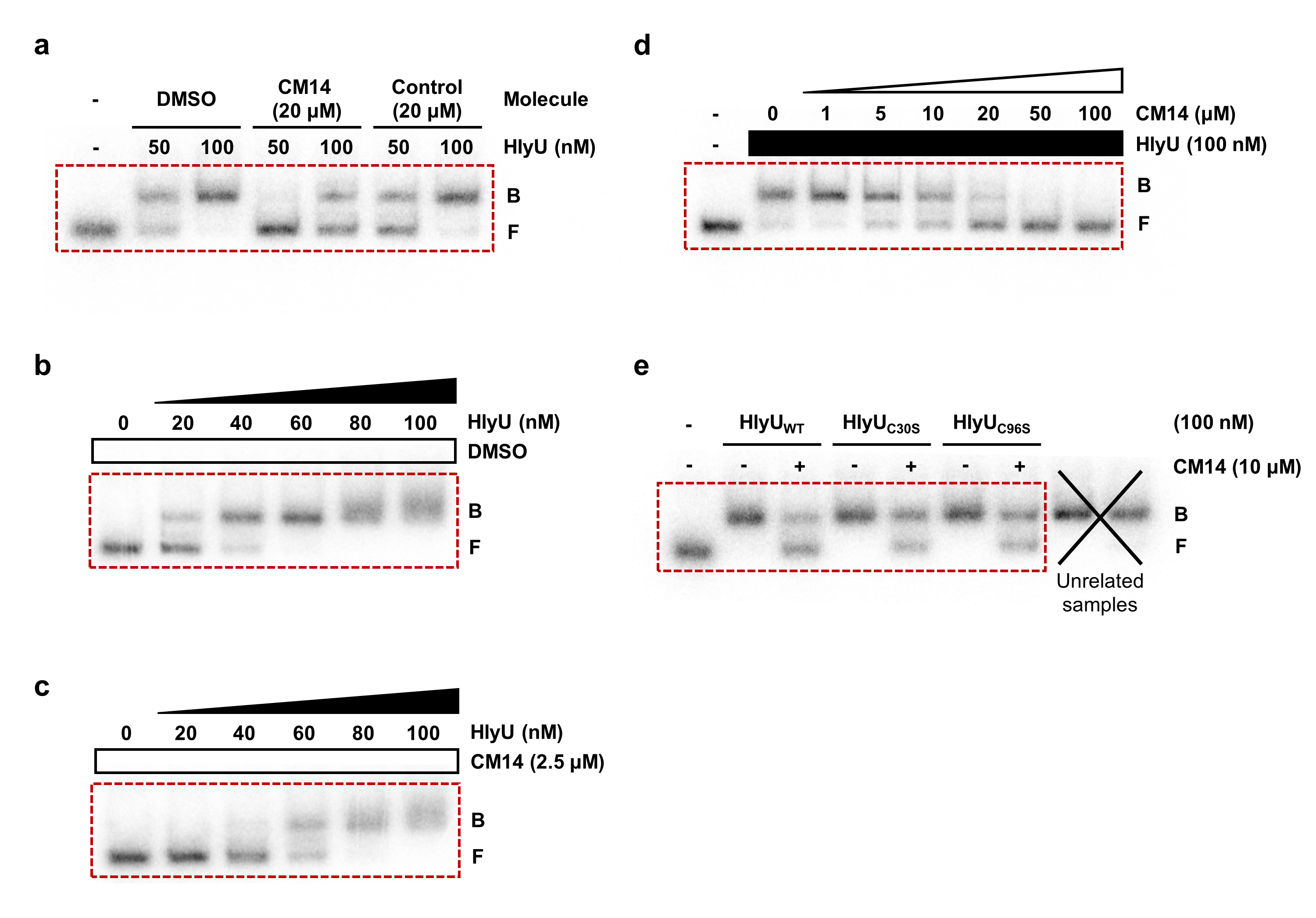

Supplement: Supplementary file 1 — Supplementary Information [file 41598_2019_39554_MOESM1_ESM.docx]
